# *Colletotrichum shisoi* sp. nov., an anthracnose pathogen of *Perilla frutescens* in Japan: molecular phylogenetic, morphological and genomic evidence

**DOI:** 10.1038/s41598-019-50076-5

**Published:** 2019-09-16

**Authors:** P. Gan, A. Tsushima, R. Hiroyama, M. Narusaka, Y. Takano, Y. Narusaka, M. Kawaradani, U. Damm, K. Shirasu

**Affiliations:** 1 RIKEN Center for Sustainable Resource Sciences, Yokohama, Kanagawa Japan; 2 Graduate School of Science, The University of Tokyo, Bunkyo, Tokyo Japan; 3 Research Institute for Biological Sciences, Okayama Prefectural Technology Center for Agriculture, Forestry, and Fisheries, Okayama, Japan; 4 Graduate School of Agriculture, Kyoto University, Kyoto, Japan; 5 Research Institute of Environment, Agriculture and Fisheries, Osaka, Japan; 6 Senckenberg Museum of Natural History Görlitz, 02806 Görlitz, Germany

**Keywords:** Fungal genomics, Fungal pathogenesis

## Abstract

Species of the fungal genus *Colletotrichum* are among the most devastating pathogens of agricultural crops in the world. Based on DNA sequence data (ITS, *GAPDH*, *CHS-1*, *ACT*, *TUB2*) and morphology, we revealed *Colletotrichum* isolates infecting the oil crop *Perilla frutescens*, commonly known as shiso, to represent a previously unknown species of the *C. destructivum* species complex and described it as *C. shisoi*. We found that *C. shisoi* appears to be able to adopt a hemibiotrophic lifestyle, characterised by the formation of biotrophic hyphae followed by severe necrotic lesions on *P. frutescens*, but is less virulent on *Arabidopsis*, compared to its close relative *C. higginsianum* which also belongs to the *C. destructivum* species complex. The genome of *C. shisoi* was sequenced, annotated and its predicted proteome compared with four other *Colletotrichum* species. The predicted proteomes of *C. shisoi* and *C. higginsianum*, share many candidate effectors, which are small, secreted proteins that may contribute to infection. Interestingly, *C. destructivum* species complex-specific secreted proteins showed evidence of increased diversifying selection which may be related to their host specificities.

## Introduction

*Perilla frutescens*, or shiso, is an herbaceous plant belonging to the Lamiaceae family and was originally cultivated throughout East and South-East Asia as a culinary herb, oil source and as a traditional medicine^1,2^. The plant produces a significant amount of volatile compounds^3^ that contribute to its distinctive odour, as well as a high amount of polyphenols with antioxidant activities^4^. Apart from direct consumption, *P. frutescens* is also used industrially, whereby oil from the seed may be used as a drying oil^2^. In Korea, perilla oil has consistently been the third highest domestically produced vegetable oil with a volume of more than 30,000 metric tonnes produced in 2015/16^5^. In Japan, 6,708 tonnes of *P. frutescens* were reported to be grown for direct consumption, while a further 2,763 tonnes were grown for industrial uses in 2014^6^. In the West, perilla has been grown as an ornamental plant since the Victorian Era, where it is known as the “beefsteak plant”^7^.

Fukui (1925) first reported an anthracnose disease of perilla in Japan and described the causal organism as *Colletotrichum yoshinaoi* Fukui^8^. In a study from Korea, the causal agents of perilla anthracnose were identified as *C. gloeosporioides, C. coccodes*, *C. dematium* and *Glomerella cingulata*, which had previously been regarded as the sexual morph of *C. gloeosporioides*^9^. In the study, the authors hypothesised the species described by Fukui to be a synonym of *C. gloeosporioides*, since *C. gloeosporioides* was more frequently isolated from infected plants^9^. However, none of these reports was confirmed by molecular data and the systematic position of *C. yoshinaoi*, which lacks a living type strain, is unknown.

More recently, *C. destructivum* was identified as being responsible for perilla anthracnose in Japan using ITS sequences^10^, while fungal strains causing anthracnose of *Lamium amplexicaule* (henbit), that also belongs to the Lamiaceae, were identified as *C. higginsianum* based on ITS sequences^11^. However, all previous reports were carried out before the epitypification of the respective species and the treatments of the respective species complexes^12–16^ and were based on morphology or ITS only.

With the advent of affordable high throughput genome sequencing technologies, the genomes of multiple members of the *Colletotrichum* genus of plant pathogenic fungi have been sequenced and released^17–27^. Sequenced genomes have included strains belonging to different species complexes, which comprise closely related species that are phylogenetically distinct from other members of the same genus. Members of the same species complex exhibit similarities in terms of their infection lifestyles and whole genome comparative analyses have revealed genomic adaptions that contribute to these differences^17,18,28^. For example, members of the *Colletotrichum graminicola* species complex which specifically infect graminiaceous hosts have reduced numbers of pectin-degrading enzymes^17,18^. Comparisons between different members of the same species complex, such as between *C. sublineola* and *C graminicola*, which infect sorghum and maize respectively, have also been performed; this led to the identification of genes that were not found to be conserved between different members of the same species complex, and which may contribute to adaptation to their specific host niches^29^.

The aims of this study were to characterise one of the causal agents of anthracnose of *P. frutescens* in Japan based on multi-locus sequence data and morphology. Further, we aimed to characterise the species at the molecular level by sequencing and assembly of its genome. In addition, we wanted to identify what types of genes show different conservation patterns between shiso-infecting *Colletotrichum* and close relatives in the *Colletotrichum* genus. In particular, we aimed to analyse the conservation patterns of genes encoding small, secreted proteins, since these may contribute to differences in infection outcomes.

## Results

### Multi-locus phylogenetic analysis

An initial BLASTn search of the NCBI non-redundant nucleotide database using the internal transcribed spacers (ITS) sequence from *Colletotrichum* strain JCM 31818 from *P. frutescens* as a query was conducted, revealing that seven of the top ten hits, differing by 7–8 mismatches, belong to the *C. destructivum* species complex (Supplementary Table S1). As strain MAFF 240106 was also isolated from *P. frutescens* and was previously identified as *C. destructivum* on the basis of its ITS sequence^10^, sequences from both strains were compared and found to be identical. In order to identify these strains to the species level, a phylogenetic tree based on ITS, glyceraldehyde-3-phosphate dehydrogenase (*GAPDH)*, chitin synthase 1 (*CHS-1*), actin (*ACT*) and beta-tubulin (*TUB2*) sequences was calculated and used for comparison of the strains from *P. frutescens* with all currently accepted species in the *C. destructivum* species complex (Supplementary Table S2). DNA sequences obtained from the MAFF Genebank project of several strains isolated from *L. amplexicaule*, a host from the same family as *P. frutescens*, which had previously been identified as *C. higginsianum* based on ITS sequences^11^, were also included (Supplementary Table S2).

In the multi-locus phylogenetic analysis, sequences were aligned, trimmed and then concatenated to generate a sequence alignment comprising 1,778 characters (gene boundaries ITS: 1–546, *GAPDH*: 547–740, *CHS-1*: 741–1,020, *ACT*: 1,021–1,275, *TUB2*: 1,276–1,778) from 94 isolates.

In maximum parsimony analyses, 1,318 characters were found to be constant, while 266 and 194 of the variable characters were found to be parsimony informative and uninformative respectively. The heuristic search yielded 64 equally most parsimonious trees (tree length: 659, consistency index (CI): 0.819, retention index (RI): 0.931, rescaled consistency index (RC): 0.763, homoplasy index (HI): 0.181). Analysis of the concatenated alignment as well as alignments of each individual gene indicated that the strains from *P. frutescens* are distinct from the other members of the *C. destructivum* species complex (Supplementary Figs 1–6) and may represent a separate species.

**Figure 1:**
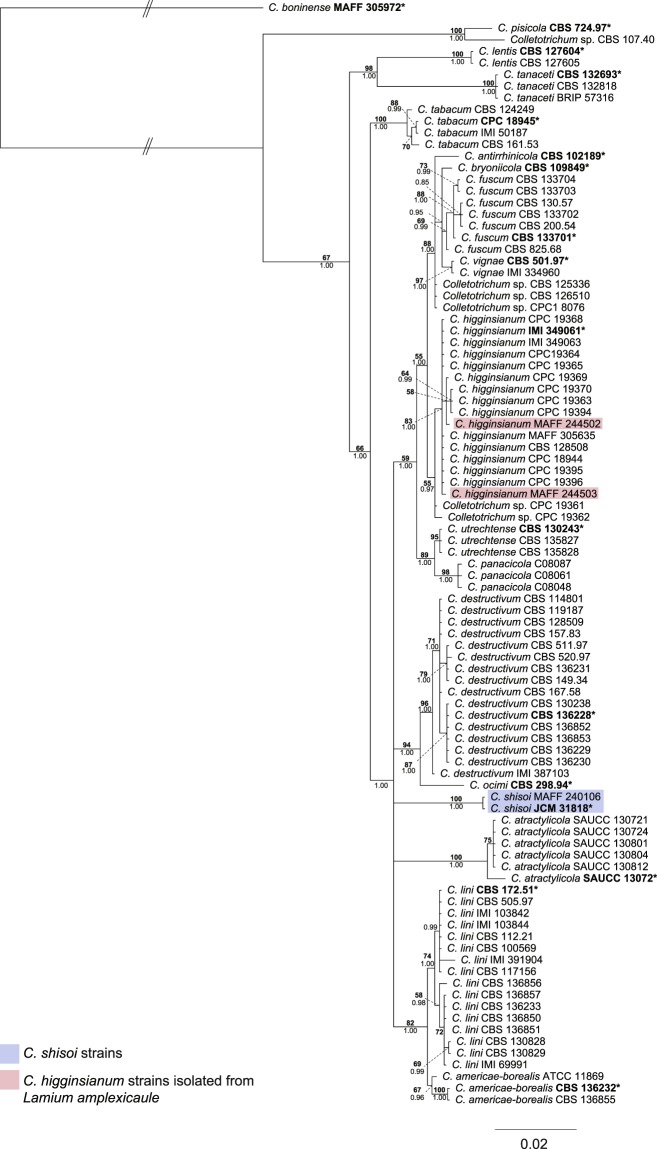
Multi-locus phylogenetic tree based on ITS, *GAPDH*, *CHS-1*, *ACT* and *TUB2* sequences of the *Colletotrichum destructivum* species complex using *Colletotrichum boninense* MAFF 305972 as an outgroup. Values at the nodes are Bayesian posterior probability values above 50%. *C. shisoi* strains are highlighted in blue. *C. higginsianum* strains isolated from *Lamium amplexicaule* are highlighted in red. Ex-type cultures are marked with an asterisk and in bold. Branches with double-bars are truncated two-fold.

To confirm this, maximum likelihood and Bayesian phylogenetic analyses were carried out. The best model for phylogenetic analysis of *ACT, CHS-1*, *GAPDH*, ITS and *TUB2* was calculated as HKY + G, K80, HKY + I, K80 + I + G and K80 + I, respectively. The consensus tree obtained from Bayesian analysis of the multi-locus alignment showed the strains from *P. frutescens* form a distinct clade on a long branch with a Bayesian posterior probability value of 1.00 (Fig. 1), while the strains from *L. amplexicaule* (MAFF 244502, 244503) clustered with *C. higginsianum*, confirming their identities as *C. higginsianum* strains. In each consensus tree of individual loci generated by Bayesian analysis (Supplementary Figs 7–11), the strains obtained from *P. frutescens* formed a distinct clade within the *C. destructivum* species complex with Bayesian posterior probability values above 0.9. However, the position of this clade containing isolates from *P. frutescens* differed depending on the locus. The topologies of the ML trees calculated from the single and multi-locus alignments were consistent with the results from the Bayesian analyses (Supplementary Figs 12–17).

### Taxonomy

Based on the DNA sequence data, the *Colletotrichum* species from *P. frutescens* was found to be distinct from other species in the *C. destructivum* species complex and therefore described as a new species below.

#### Colletotrichum shisoi

**Figure 2:**
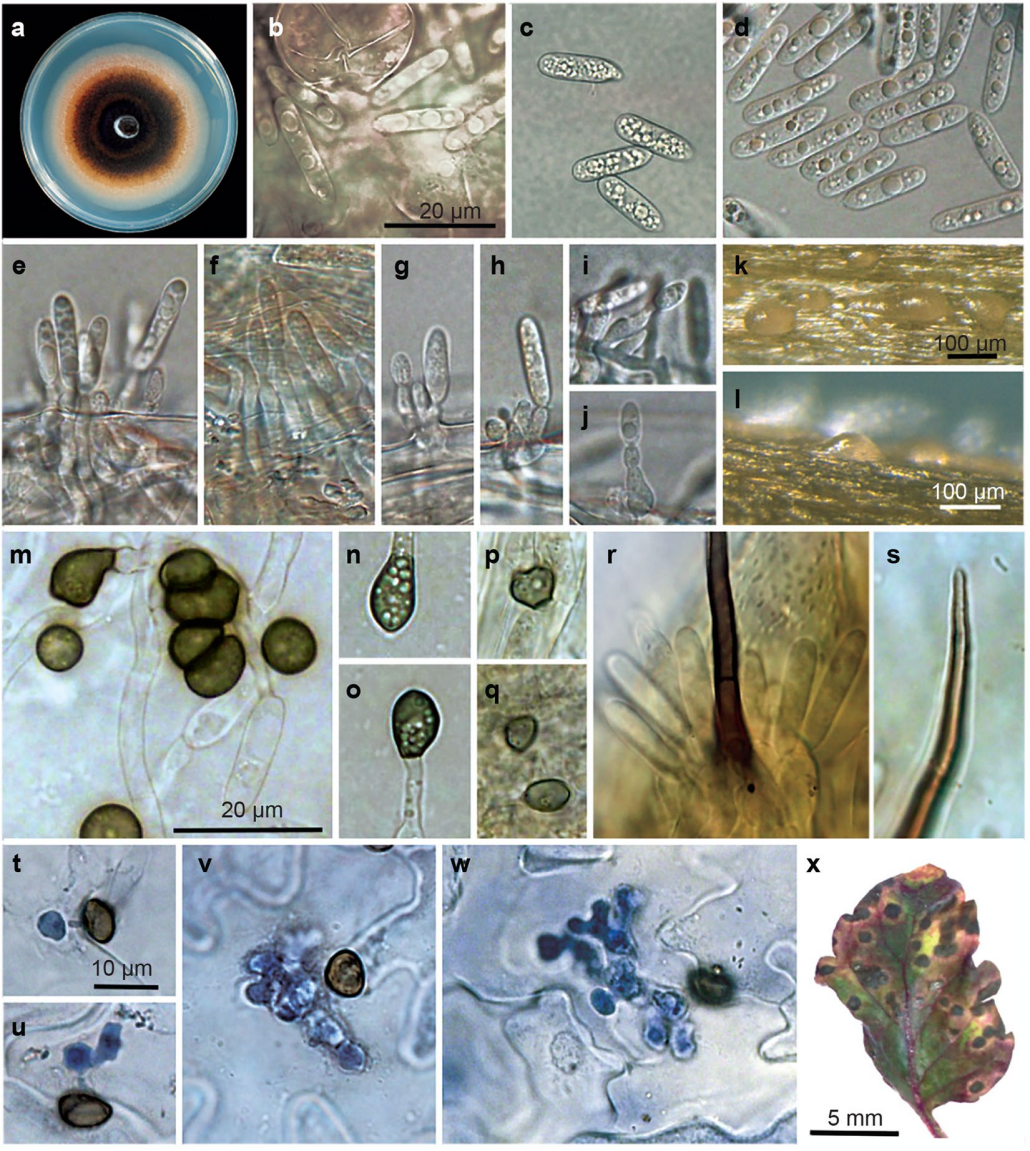
*Colletotrichum shisoi* (ex-holotype strain JCM 31818). (**a**) 10-day old culture on PDA. (**b**–**d**) Conidia from *P. frutescens* leaves, PDA and SNA, respectively. (**e**–**j**) Conidiophores on *Anthriscus sylvestris* stems. (**k**–**l**) Conidiomata on *A. sylvestris* stems. (**m**). Appressoria formed on OA. (**n**–**o**) Appressoria formed on SNA. (**p**–**q**) Appressoria formed on *A. sylvestris* stems. (**r**–**s**) Setae formed on *P. frutescens* leaves. (**t**–**u**) Trypan blue stained *C. shisoi* growing in perilla leaves at 40 hpi. (**v–w**) Biotrophic hyphae at 60 hpi in infected *P. frutescens* leaves. (**x**) Symptoms on *P. frutescens* leaves at two weeks after inoculation with *C. shisoi*. Scale bar of (**b**) applies to (**e–j**); scale bar of (**m**) applies to (**n–s**) and scale bar for (**t**) applies to (**u–w**).

P. Gan, A. Tsushima, M. Kawaradani, Damm & K. Shirasu sp. nov., Mycobank MB 828333, Fig. 2.

#### Etymology

Refers to the host from which the species was isolated, *Perilla frutescens* var. *crispa*, commonly known as shiso.

*Sexual morph* not observed. *Asexual morph on synthetic nutrient-poor agar* (*SNA). Vegetative hyphae* 3–5.5 μm, average (av.) ± standard deviation (SD): 4.0 ± 1.0 µm diam, hyaline, smooth-walled, septate and branched. *Chlamydospores* not observed. *Conidiomata* absent, conidiophores formed directly on conidiogenous cells. *Setae* not observed *Conidiogenous cells* hyaline to pale brown, smooth-walled, septate, cylindrical. *Conidia* hyaline, smooth-walled, aseptate, cylindrical, straight, with both ends round or with one end slightly tapered, 13–22.5 µm × 3–5 µm, av. ± SD: 16.5 ± 2.5 µm diam × 4.0 ± 0.5 µm, L/W ratio: 4.5. *Appressoria* single, olivaceous black, smooth-walled, obovoid to clavate with truncate base, 5.5–10 µm × 4–7.5 µm, av. ± SD: 7.5 ± 1.0 µm diam × 6.0 ± 1.0 µm, L/W ratio: 1.5.

*Asexual morph on oatmeal agar (OA)*. *Vegetative hyphae* 1.5–4.0 μm diam, hyaline, smooth-walled, septate and branched. av. ± SD: 2.9 ± 0.5 µm diam. *Setae* unbranched, medium brown, smooth-walled, 1–2 septate, 29.5–76.0 µm long, tip rounded. *Conidia* 12–23 µm × 3.5–5.5 µm, av. ± SD: 16.0 ± 3.0 × 4.5 µm ± 0.5 µm, L/W ratio: 3.5. *Appressoria* single or in groups, greenish olivaceous to olivaceous buff, smooth-walled, globose to subglobose, 5.5–11 µm × 4.5–10 µm, av. ± SD: 8 ± 1.0 µm diam × 6.5 ± 1.0 µm, L/W ratio: 1.0.

*Asexual morph on PDA. Vegetative hyphae* 2.0–7.0 μm diam, hyaline, smooth-walled, septate and branched. av. ± SD: 4.0 ± 1.0 µm diam. *Conidia* 15.0–27.0 µm × 3.0–5.0 µm, av. ± SD: 17.0 ± 2.0 µm × 4.0 ± 0.0 µm, L/W ratio: 4.0. *Setae* unbranched, medium brown, smooth-walled, 1–2-septate, 23.5–78.0 µm in length, tip rounded. Appressoria 7.0–11.0 µm × 5.0–8.0 µm, av. ± SD: 9.0 ± 1.0 µm × 7 ± 1.0 µm, L/W ratio: 1.0.

*Asexual morph on autoclaved Anthriscus stem. Conidiomata* conidiophores formed on hyaline cells. *Setae* not observed. *Conidiophores* hyaline, smooth-walled, simple. *Conidiogenous cells* hyaline, smooth-walled, straight with round ends or slightly tapered at one end. *Conidia* hyaline, smooth-walled, aseptate, cylindrical, straight to slightly curved, with both ends straight or with one end tapered 15.5–28.5 µm × 3.0–5.0 µm, av. ± SD: 19.0 ± 3.0 µm × 4.0 ± 0.5 µm, L/W ratio: 5.0. *Appressoria* single or in loose groups, globose to subglobose, occasionally with an irregular in shape, 3.0–11.0 µm × 3.5–7.5 µm, av. ± SD: 6.5 ± 2.0 µm × 5.0 ± 1.0 µm, L/W ratio: 1.0.

*Asexual morph, infection structures and symptoms in/on leaves of* Perilla frutescens: Lesions on leaves small, elliptical or irregular, appearing on cotyledons and fully developed leaves, gradually enlarging and becoming dark brown. Acervuli observed forming on lesions under conditions of high humidity. *Setae* unbranched, medium brown, smooth-walled, 1–4-septate, 77.5–45.5 µm long and 3–8.5 µm diameter in the base, base ampulliform or cylindrical, tip rounded. *Conidia* hyaline, smooth-walled, aseptate, cylindrical, straight to very slightly curved, with ends round or with one end tapered, 11.0–21.5 µm × 3.0–5.0 µm, av. ± SD: 17.0 ± 2.0 µm × 4.0 ± 0.5 µm, L/W ratio: 4.5. *Appressoria* single or in loose groups, 4.0–8.0 µm × 3.0–6.0 µm, av. ± SD: 6.0 ± 1.0 µm × 4.5 ± 1.5 µm, L/W ratio: 1.5. *Intracellular hyphae in detached* Perilla frutescens *leaves*: bulbous, hyphae observed within penetrated cells from 40 hpi.

#### Culture characteristics

Colonies on SNA at 25 °C flat with entire margin, hyaline, filter paper and *Anthriscus* stem partly covered with salmon and dark chestnut acervuli. Whitish aerial mycelia on medium. Reverse same colors. Growth rate 34–39 mm in 7 d (48–51.5 mm 10 d). Colonies on OA flat, radially striate with lobate edge, reverse the same, Growth rate 36.5–37.5 mm in 7 d (51.5–53 mm 10 d), olivaceous brown to brick, with white aerial hyphae at the edge, colonies of strain MAFF 240106 differ in forming radial crinkles, Colonies on PDA. Flat, olivaceous brown to light orange, hyaline at the edge. Growth rate 46.5–51 mm in 7 d (68–71 mm 10 d). *Conidia* in mass saffron.

#### Materials examined

JAPAN, Osaka, Ibaraki City from lesions of cultivated *Perilla frutescens* var. *crispa* cv. Aka-shiso, collection date 1 August 2006, collected by M. Kawaradani (TNS-F-40462 holotype, culture ex-holotype JCM 31818); JAPAN, Osaka, Ibaraki City from lesions of cultivated *Perilla frutescens* var. *crispa* cv. Aka-shiso, collection date July 2006, collected by M. Kawaradani (MAFF 240106). MAFF 240106 was characterised as being able to infect green and red shiso; as well as egoma varieties of *Perilla frutescens* from Japan, with strongest symptoms on red shiso^10^.

Notes: *Colletotrichum shisoi* is only known from *P. frutescens* plants in Japan. It belongs to the *C. destructivum* species complex and can be identified by its ITS, *ACT, CHS-1, GAPDH, TUB2* sequences. Fukui (1925) reported a new anthracnose disease of *P. frutescens* in Japan caused by *C. yoshinaoi*^8^. Kim *et al*. (2001) regarded the name *C. yoshinaoi* as invalid because both a Latin diagnosis and the indication of a type is lacking^9^. However, a Latin diagnosis was only required between 1 January 1935 and 31 December 2011, and an indication of a type is only mandatory from 1 January 1958 (Art. 37.1)^30^; *C. yoshinaoi* is therefore validly described. Conidia of *C. yoshinaoi* were described as being oval with round ends and sometimes slightly curved, measuring 15–17 × 4–5 µm with an L/W ratio = 4, which is overlapping with *C. shisoi*. However, setae of *C. yoshinaoi* measure 40–50 × 3 µm and are sometimes slightly curved and appressoria are round (corresponding to L/W ratio of 1) and about 6 µm diam, while setae of *C. shisoi* are larger, measuring 45.5–77.5 × 3–8.5 µm and are straight and appressoria of *C. shisoi* on the host plant measure 4–8 µm × 3–6 µm with a L/W ratio of 1.5. Moreover, *C. yoshinaoi* infects stems causing early defoliation and inhibits fruiting and was never observed on leaves^8^, whereas *C. shisoi* infects cotyledons and fully developed leaves. Therefore, Kawaradani (2008) did not regard strain MAFF 240106 (included in this study as *C. shisoi*) as *C. yoshinaoi* but identified it as *C. destructivum*. Consequently, we describe the species in the *C. destructivum* complex infecting perilla leaves as a new species, instead of epitypifying *C. yoshinaoi*.

Another *Colletotrichum* species, *C. perillae*, causes a similar disease as *C. yoshinaoi* on stems and pedicels of *P. ocymoides* in the Primorskaya and Ussurskaya Oblast, an area in Russia close to Japan. This species forms acervuli with straight, cylindrical conidia with rounded ends, measuring 18–22 × 4.5–6 µm and straight to flexuous, aseptate, olivaceous setae becoming paler towards the tip, measuring 43–48 × 4–5 µm^31^. Apart from the fact that the disease caused is different, the conidia of *C. perillae* are larger than those of both *C. shisoi* (and *C. yoshinaoi*) and setae are shorter and possibly also differ in septation from those of *C. shisoi*. This species was also described without a Latin diagnosis, but before 1 January 1935; the name is therefore invalid (Art. 39.1)^32^.

### Pathogenicity tests

**Figure 3:**
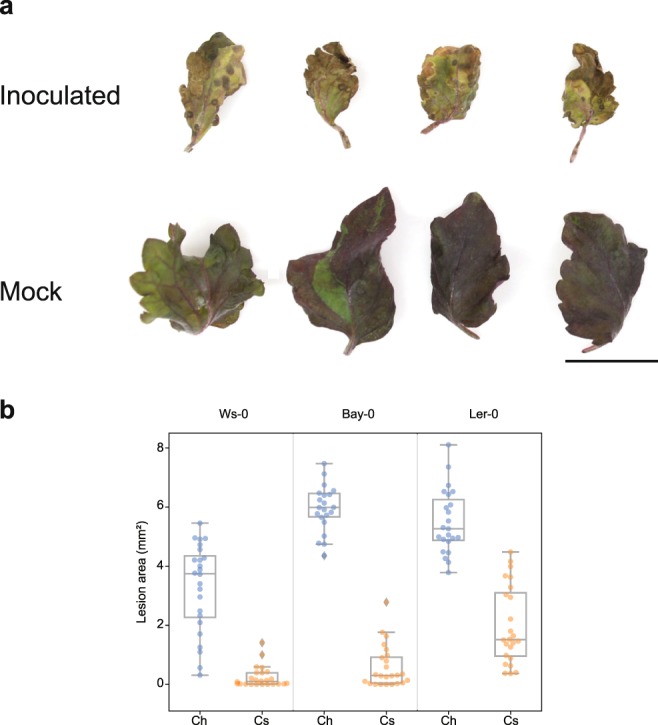
Pathogenicity of *Colletotrichum shisoi*. (**a**) *Perilla frutescens* var. *crispa* Aka-shiso leaves inoculated with *C. shisoi* (top) compared to mock inoculated leaves (bottom) at two weeks post inoculation. Scale bar = 1 cm. The same results were observed in three independent experiments. (**b**) Boxplots comparing area sizes of lesions formed on leaves of *Arabidopsis thaliana* ecotypes after inoculations with *C. shisoi* (Cs) and *C. higginsianum* (Ch). The distributions of lesion areas were found to be significantly different between *A. thaliana* leaves inoculated with *C. shisoi* compared to leaves inoculated with *C. higginsianum* in all three *A. thaliana* ecotypes according to Mann-Whitney *U* tests (P-values < 0.01). Significant differences were detected in two independent experiments.

Three-week-old intact *P. frutescens* plants spray-inoculated with *Colletotrichum shisoi* JCM 31818 displayed typical symptoms of anthracnose lesions two weeks after inoculation while mock inoculated plants showed no symptoms (Fig. 3a). Symptoms were similar to symptoms of perilla anthracnose observed in nurseries of cultivated Aka-shiso *P. frutescens* plants previously reported by Kawaradani *et al*.^10^. Infected plants had smaller leaves than mock inoculated plants (Fig. 3a). These differences were reproduced in three independent experiments. The same fungus was consistently re-isolated from lesions of inoculated plants.

As *C. shisoi* is closely related to the *Arabidopsis thaliana*-infecting species *C. higginsianum*, we tested if it can infect *A. thaliana*. *C. shisoi* did not form lesions on *A. thaliana* ecotypes Bay-0 and Ws-0 but could form lesions on Ler-0, although to a lesser extent than *C. higginsianum* (Fig. 3b). The distributions of lesion areas were found to be significantly different between *C. shisoi* and *C. higginsianum* with P-values < 0.01 according to Mann-Whitney *U* tests in all three ecotypes.

### Genome sequence analysis

**Figure 4:**
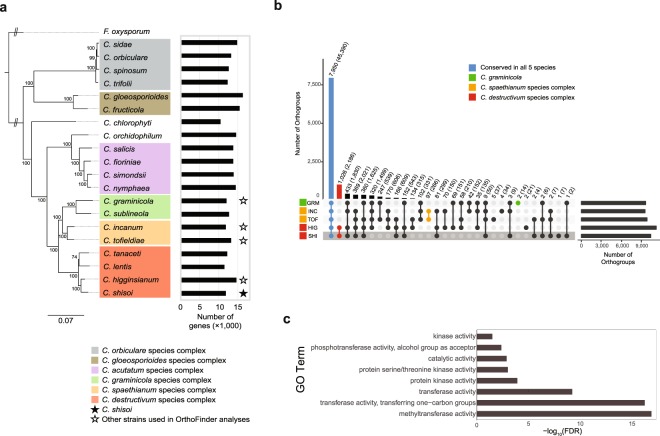
Conservation of all predicted proteins in selected *Colletotrichum* species. (**a**) Maximum likelihood phylogeny based on 128,141 characters from 254 eukaryotic conserved single copy protein orthologs identified by BUSCO analysis in different members of the *Colletotrichum* genus, showing the relationship between *C. shisoi* and other *Colletotrichum* species complex members. Predicted gene numbers are indicated by the bars on the right. *Fusarium oxysporum* was used as an outgroup. Numbers at the nodes represent percent support values out of 500 bootstrap replicates. (**b**) UpSet plot showing conservation of protein orthogroups in *C. graminicola* (GRM), *C. incanum* (INC), *C. tofieldiae* (TOF), *C. higginsianum* (HIG) and *C. shisoi* (SHI). Box plots show the distributions of orthogroup protein length means. Bars represent number of orthogroups showing specific conservation patterns with numbers also given above each bar. Numbers in brackets show number of proteins that belong to orthogroups according to their respective conservation pattern. (**c**) Bar chart showing GO terms significantly enriched among *C. shisoi* genes present only in the *C. destructivum* species complex (*C. shisoi* and *C. higginsianum*). FDR: False discovery rate of hypergeometric test after Benjamini-Hochberg multiple testing correction.

The genome size of *C. shisoi* was estimated, according to k-mer analysis, to be 58.6 Mb and sequenced to 603 × coverage. A total of 36,350 contigs were assembled from the 100 bp paired-end libraries with an N50 value of 7,997. These were then assembled into 20,745 scaffolds with N50 of 9,321 bp (Table 1). According to BUSCO analysis, 98.3% of 3,725 sordariomycete conserved proteins could be identified as complete sequences within the assembly, with an additional 0.8% found to be fragmented, indicating coverage of most of the gene coding space (Table 1). A total of 11,848 genes were predicted in the *C. shisoi* genome. The number of genes encoded is consistent with the gene numbers predicted in other *Colletotrichum* species (Fig. 4a), whose numbers range from 16,287 genes (*C. gloeosporioides*) to 10,419 (*C. chlorophyti*).

**Table 1:** Genome assembly statistics.

Assembly	
Coverage	603×
Number of scaffolds (>=0 bp)	20,745
Number of scaffolds (>=1,000 bp)	9,321
Largest scaffold	153,238 bp
Total length	69.7 Mb
Total length of scaffolds (>=1,000 bp)	63.87 Mb
GC (%)	47.14
N50	15,836 bp
N75	4,863 bp
L50	1,051
L75	2,981
Number of N’s per 100 kbp	943.26
Complete	98.3%
Fragmented	0.8%
Duplicated	0.1%

Complete: Percentage of BUSCO sordariomycete_odb9 conserved single copy gene set that was present as a complete coding sequence in the assembly; Fragmented: percentage of BUSCO sordariomycete_odb9 conserved single copy gene set that were identified as partial coding sequences in the assembly; Duplicated: percentage of BUSCO sordariomycete_odb9 single copy gene set that was found to be present with more than one copy in the assembly.

### Conservation of proteins amongst Colletotrichum species

**Figure 5:**
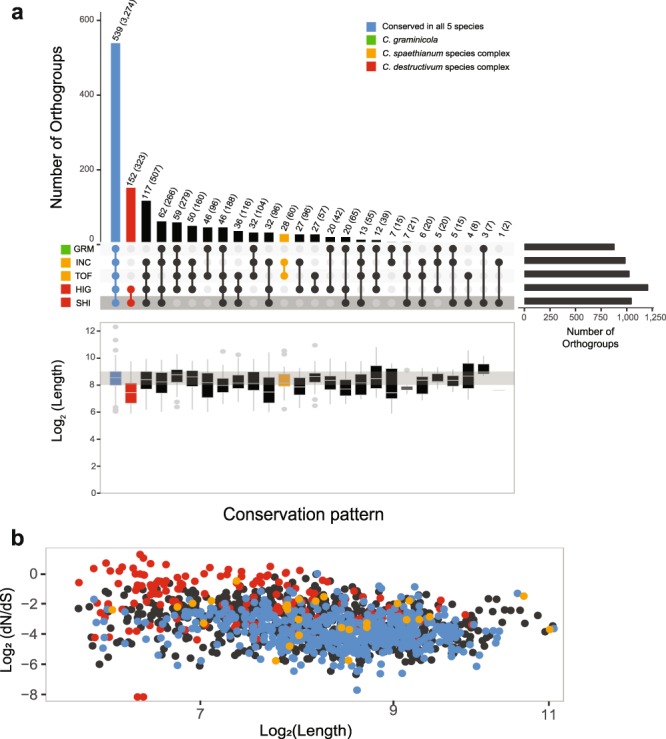
Conservation of secreted proteins in *Colletotrichum* species. (**a**) UpSet plot showing conservation of secreted protein orthogroups in *C. graminicola* (GRM), *C. incanum* (INC), *C. tofieldiae* (TOF), *C. higginsianum* (HIG) and *C. shisoi* (SHI). Box plots show the distributions of orthogroup protein length means. Bars represent number of orthogroups showing specific conservation patterns with numbers also given above each bar. Numbers in brackets show number of proteins that belong to orthogroups according to their respective conservation pattern. (**b**) Scatterplot showing orthogroup protein length means relative to the mean rate of pairwise non-synonymous mutations (dN)/synonymous mutations (dS) in each orthogroup.

The conservation of genes between *C. shisoi* and four closely related and sequenced *Colletotrichum* species was assessed (Figs 4 and 5). The four that were chosen for comparisons were *C. higginsianum*, which has a chromosome-level assembly, publicly available annotations and also belongs to the *C. destructivum* species complex, *C. incanum* and *C. tofieldiae*, from the *C. spaethianum* species complex, and *C. graminicola* from the *C. graminicola* species complex. Members of the *C. spaethianum* and the *C. graminicola* species complexes were selected since they are closely related to the *C. destructivum* species complex (Fig. 4a). Among the five species assessed, *C. higginsianum* encodes the greatest number of predicted genes (14,651 genes). The number of predicted proteins for the other species were closer to the number of genes in *C. shisoi* with 11,436, 12,501 and 12,006 proteins predicted in *C. incanum, C. tofieldiae* and *C. graminicola*, respectively (Fig. 4a). A total of 11,914 orthogroups with two or more proteins were identified (Fig. 4b). Of these, 7,950 groups (74.0% proteins from *C. shisoi*, 63.0% from *C. higginsianum*, 78.3% from *C. incanum*, 74.2% from *C. tofieldiae* and 73.7% from *C. graminicola*) were conserved in all five species (Supplementary Tables S3 and S4). From this analysis, all *C. shisoi* genes could be classified into an orthogroup with a related sequence in one of the four other species or in the same genome. Only one orthogroup was predicted to be *C. shisoi-*specific. This orthogroup consisted of seven proteins annotated as MFS transporter proteins. Similarly, all *C. higginsianum* proteins were classified into an orthogroup with only two orthogroups found to be specific to *C. higginsianum*, one consisting of 13 ABC transporter genes and the second, consisting of 8 secondary metabolite regulator laeA protein-encoding genes. As expected from their close evolutionary relationship, *C. shisoi* and *C. higginsianum* were found to share an additional 2,585 orthogroups consisting of 23.4% proteins from *C. shisoi* and 20.8% proteins from *C. higginsianum*, including 1,026 orthogroups (8.7% proteins from *C. shisoi* and 7.4% proteins from *C. higginsianum*), which are present only in these two members of the *C. destructivum* clade. Proteins of *C shisoi* from the *C. destructivum*-specific orthogroups were significantly enriched for Gene Ontology (GO) terms involved in methyltransferase and protein kinase activity (FDR < 0.05) (Fig. 4c). In contrast, *C. tofieldiae* and *C. incanum*, which both belong to the *Colletotrichum spaethianum* clade, share only 97 orthogroups, consisting of 1.3% proteins from *C. incanum* and 0.9% proteins from *C. tofieldiae*, which were specific to these two members of the *C. spaethianum* clade.

### Conservation of secreted proteins

As secreted proteins are known to be important for infection, their conservation between the five species was also assessed (Fig. 5a, Supplementary Table S5). A total of 1,360 secreted protein orthogroups were identified. Of these, 540 orthogroups (39.7%) were found to be conserved in all five species. These included 48.4% proteins out of the 1,274 predicted secreted proteins from *C. shisoi* and 40.6% proteins of the 1,644 predicted secreted proteins from *C. higginsianum* (Supplementary Table S5). A further 154 secreted protein orthogroups, were identified as specific to the two *C. destructivum* clade members. In contrast, 28 secreted protein orthogroups were identified as being *C. spaethianum* clade-specific. No GO terms were found to be significantly associated with *C. shisoi* secreted proteins that were in *C. destructivum* clade-specific orthogroups. Since effector proteins tend to be small, secreted proteins under positive selection, we plotted the average lengths of orthogroups consisting of secreted proteins (Fig. 5a) to investigate if there was a relationship between conservation pattern and orthogroup protein length. All 2,186 *C. destructivum* clade-specific proteins (Fig. 4b) were found to be shorter than proteins belonging to orthogroups that were conserved in the five *Colletotrichum* species. Further, of particular interest to this study, the *C. destructivum* clade-specific secreted proteins (Fig. 5a) were found to be also under higher rates of positive selection, with higher rates of non-synonymous to synonymous mutations (dN/dS), compared to secreted proteins that were conserved in all five tested *Colletotrichum* species (Fig. 5b). In contrast, this was not observed among *C. spaethianum* clade-specific secreted proteins (Fig. 5b). No species-specific groups were identified amongst the secreted protein orthogroups in all five species, indicating that species-specific sequences did not belong to multi-gene families. A total of 846 secreted proteins, consisting of 216 proteins from *C. graminicola*, 128 proteins from *C. incanum*, 112 proteins from *C. tofieldae*, 225 proteins from *C. higginsianum* and 135 proteins from *C. shisoi* were not assigned to any orthogroup, and were found to be species-specific (Supplementary Table S5).

## Discussion

*Colletotrichum* species can infect a wide range of plants. In this study, we identified a new species in the *C. destructivum* clade that infects the commercially important oil crop *P. frutescens*. Previously, species of the *C. gloeosporioides* clade, *C. gloeosporioides, C. dematium* and *C. coccodes* were reported as pathogens of *P. frutescens* in Korea by morphological examination of isolates^9^, and *C. destructivum* was identified as a pathogen of *P. frutescens* in Japan based on ITS sequences^10^. In this study, a multi-locus phylogenetic analysis showed that strains from *P. frutescens* previously identified as *C. destructivum*, are genetically distinct from other known species of the *C. destructivum* species complex, and were thus described as a new species, *C. shisoi*. Since well-studied species of the *C. destructivum* species complex have previously been confused with *C. coccodes*, *C. gloeosporioides and Glomerella cingulata*^12^, the strains from *P. frutescens* in Korea^9^ identified as these species could also represent *C. shisoi* and their re-examination may be warranted.

In order to characterise *C. shisoi* and to allow comparisons to other members of the *Colletotrichum* genus at the molecular level, the genome of *C. shisoi* was sequenced and assembled. The size of the *C. shisoi* assembly is 69.7 Mb, which is larger than those of other sequenced members in the *C. destructivum* species complex, including *C. higginsianum*^33^ (50.72 Mb), *C. lentis*^21^ (56.1 Mb) and, most recently, *C. tanaceti*^26^ (57.9 Mb). These sizes are closer to the genome size of *C. shisoi* estimated by k-mer analysis (58.6 Mb). A genome assembly size may deviate from k-mer estimates due to high levels of repeats or heterozygosity^34^. Members of the *C. destructivum* clade are known to be haploid pathogens that propagate asexually and thus heterozygosity is unlikely^12^. Further, BUSCO analysis of conserved genes reveals that only 0.1% of the conserved coding sequences present in the genome are duplicated, indicating that the genome is not heterozygous and that the gene coding regions at least are likely not to be duplicated. This is consistent with the predicted number of genes (11,848) in *C. shisoi*, which is within the range of other sequenced members of the *C. destructivum* species complex including the recently published genomes of *C. lentis*^21^ (11,436), *C. tanaceti*^26^ (12,172) and another isolate of *C. higginsianum*^25^ (MAFF 305635-RFP), which has 12,915 protein coding genes. It is noted that the genome assembly of *C. shisoi* generated in this study was sequenced using only short reads and is highly fragmented, with more than half of the scaffolds (11,424 scaffolds totalling 5.73 Mb) being less or equal to 1 kb in length. An earlier version of the genome of its close relative, *C. higginsianum*, which was assembled using a combination of Illumina GAII, Roche 454 and Sanger Fosmid reads, also suffered from fragmentation (10,269 scaffolds)^18^, possibly due to the abundance of transposable element-rich genomic regions. Since then, a chromosome level assembly for *C. higginsianum* has been generated using a combination of PacBio long reads and optical mapping data^33^. The *C. shisoi* genome may similarly be better resolved by adopting a similar sequencing strategy.

Comparison of the genomes of *C. shisoi* and *C. higginsianum* showed that the majority of *C. shisoi* genes (89%) have orthologous sequences in *C. higginsianum*. The latter species was previously reported^11^ to infect *L. amplexicaule*, a plant belonging to the Lamiaceae family. Both pathogens appear to adopt similar infection strategies. *C. shisoi* was observed to form bulbous intracellular hyphae within infected epidermal cells in early infection of *P. frutescens* leaves. These hyphae are morphologically similar to the primary, biotrophic hyphae formed by *C. higginsianum* infecting *A. thaliana* leaves^35^. Further, as in the case of *C. higginsianum-*infected *A. thaliana* plants, necrotic lesions formed later in infection. Taken together, these observations suggest that *C. shisoi* also adopts a hemibiotrophic infection strategy, as do other members of the *C. destructivum* complex^36^.

Previously, genus-wide analyses including members from the *C. orbiculare, C. acutatum, C. graminicola, C. gloeosporioides* and *C. destructivum* species complexes of *Colletotrichum* revealed that *C. higginsianum* has the highest number of lineage-specific genes amongst the genomes tested^2^. However, at the time, *C. higginsianum* was the only *C. destructivum* species complex member whose genome had been sequenced. Further, the assembly was highly fragmented, leading to the possibility that gene numbers were inflated. Since then, a chromosome level assembly for *C. higginsianum* has been published^33^ and our results indicate that *C. higginsianum* and *C. shisoi* do indeed have a high number of orthogroups specific to these two *C. destructivum* species complex members. The *C. destructivum* clade-specific genes were significantly enriched in kinases, indicating the presence of *C. destructivum* clade-specific signalling pathways. It is interesting to note that *C. spaethianum* clade members, despite their close phylogenetic relationship to the *C. destructivum* species complex, do not exhibit the same expansion in lineage-specific genes.

Secreted proteins that were specific to the two members of the *C. destructivum* species complex analysed in this study were also found to be subject to higher rates of diversifying selection than secreted proteins that were identified as *C. spaethianum* clade-specific. *C. shisoi* is less virulent on *A. thaliana* compared to *C. higginsianum*, forming significantly smaller lesions or no lesions at all on the accessions tested. Interestingly, the more distantly related strains from the *C. spaethianum* species complex, *C. incanum* and *C. tofieldiae*, have both been shown to infect *A. thaliana* plants^17,37^, indicating that components required for successful invasion of *A. thaliana* were possibly present in the ancestor of the *C. destructivum* and *C. spaethianum* clades. Given that small, secreted proteins known as effectors, which are important for manipulation of hosts, are often under diversifying selection to avoid recognition by specific host immune components^38^, the *C. destructivum* clade-specific secreted proteins could be candidate effectors involved in infection of host plants and their diversification may have resulted in the differences in the observed infection outcomes of *C. higginsianum* and *C. shisoi*.

Finally, *Perilla frutescens* produces a range of antimicrobial compounds and has been characterised by transcriptomic and metabolomic analyses^39^. The examination of the genome of its pathogen, *C. shisoi*, will provide insights into the mechanisms of this pathogen to overcome host defence and thus enable the development of better control strategies.

## Materials and Methods

### Isolates

The strains studied here originate from leaves of *Perilla frutescens* with anthracnose symptoms that had been collected in August 2006 and July 2006 from a perilla seedling bed in Ibaraki city, Osaka, Japan as previously described^10^. As described by Kawaradani *et al*. (2016), the seedling bed was located in the shaded part of a southwestern-facing mountain slope. Leaves showing symptoms were surface sterilised with sterile water and incubated on PDA plates containing 100 ppm streptomycin at 25 °C^10^. Isolates were isolated by hyphal tipping^10^. The holotype of the new species was deposited in the mycological herbarium of the National Museum of Nature and Science (TNS-F-40462), Tsukuba, Japan and the ex-type culture in the Japan Collection of Microorganisms (JCM 31818), Tsukuba, Japan. Isolates were stored as glycerol stocks at −80 °C and revived by incubation on PDA at 24 °C in the dark prior to experiments.

### Phylogenetic analyses

Sequences of *ACT, CHS-1, GAPDH*, ITS and *TUB2* were identified from the JCM 31818 assembly by BLASTn searches with sequences from *C. higginsianum* IMI 349063 and selecting sequence regions with the lowest E-values. The ITS sequence was used to query the NCBI non-redundant nucleotide database using default BLASTn settings to identify closely related fungal species.

Sequences for MAFF 240106 ITS, *GAPDH*, *CHS-1*, *ACT* and *TUB2* were amplified using the primer pairs ITS-1F^40^ + ITS-4^41^, GDF1 + GDR1^42^, CHS-354R + CHS-79F, ACT-512F + ACT-783R^43^ and T1^44^ + Bt-2b^45^. PCR was carried out in a thermocycler using 2 × PCR Taq polymerase mix (Promega) at 95 °C for 3 min, followed by 35 cycles of 95 °C for 30 s, 55 °C for 30 s and 72 °C for 1 min and a final extension step at 72 °C for 5 min. Phylogenetic trees were calculated as previously described^46^. Sequences of each locus (Supplementary Table S2) were aligned in MAFFT v7.215^47^ using the auto setting and trimmed using trimAl v1.2rev59^48^ using the automated1 setting. Maximum parsimony analyses were carried out with PAUP* (Phylogenetic Analysis Using Parsimony) version 4.0a (build 165)^49^ using a heuristic search of 100 random sequence additions with tree bisection and reconstruction (TBR) as the branch-swapping algorithm. All sites were treated as unordered and equally weighted with gaps treated as missing data. A total of 1,000 bootstrap replicates using the same settings were carried out to determine support for the trees. To determine the best model for analyses, jModelTest2^50^ was run on alignments with BIC criterion. For single locus trees, maximum likelihood trees were calculated using RAxML-ng using the specified jModelTest2 model with 1,000 bootstrap replicates. For Bayesian inference phylogenies based on single loci were calculated twice using MrBayes (v3.2.1) with 5 × 10^6^ generations, sampling every 1,000 generations. Under these settings, the average standard deviation of split frequencies was found to be 0.006037 for *ACT*, 0.007120 for *CHS-1*, 0.005246 for *GAPDH*, 0.007322 for ITS and 0.006178 for *TUB2*. For multi-locus sequence analysis, the trimmed alignments were concatenated and then a maximum likelihood tree was calculated with RAxML-ng^51^ using the specified jModelTest2 model for each partition with 1,000 bootstrap replicates. Bayesian inference of the concatenated alignment was calculated twice using MrBayes (v3.2.1)^52^ with 5 × 10^6^ generations, sampling every 1,000 generations. Under these settings, the standard deviation of split frequencies was 0.004981 and performance scale reduction factors were close to 1.000 for all tested parameters. The first 25% generations were discarded as burnin. Phylogenetic trees were generated for individual loci using the calculated jModelTest2 models as well as for the concatenated alignment using *C. boninense* as an outgroup.

For the genus-wide maximum likelihood tree of selected *Colletotrichum* species, BUSCO was run on the genome assemblies of *C. trifolii* (RYZW01000000)*, C. sidae* (QAPF01000000)*, C. orbiculare* (AMCV02000000) and *C. spinosum* (QAPG01000000)^24^, *C. fructicola* (ANPB00000000.1)^19^, *C. gloeosporioides* (QFRH00000000)^27^, *C. chlorophyti* (MPGH01000000)^53^*, C. orchidophilum* (MJBS00000000.1)^54^*, C. salicis* (JFFI00000000.1)*, C. fioriniae* (JARH00000000.1)*, C. simondsii* (JFBX00000000.1)*, C. nymphaea* (JEMN00000000.1)^28^*, C. sublineola* (JMSE00000000.1)^29^*, C. graminicola* (ACOD00000000.1)^18^*, C. incanum* (JTLR01000000)^17^*, C. tofieldiae* (LFIV01000000)^37^*, C. lentis* (NWBT01000000)^21^*, C. tanaceti* (PJEX00000000)^26^*, C. higginsianum* (LTAN01000000)^33^ and *Fusarium oxysporum* (GCF_000149955.1)^55^ to identify highly conserved, single copy eukaryote genes (eukaryote_odb9). Sequences of orthologs from 254 single copy genes that were identified as non-duplicated in all the tested genomes were aligned using MAFFT and trimmed using trimAl as described above. Modeltest-ng v0.1.5 (https://github.com/ddarriba/modeltest) was run to determine the best model for amino acid substitutions under BIC criterion (Supplementary Table S6). Sequences for all 254 single copy genes were concatenated and RAxML-ng was used to estimate the maximum likelihood phylogeny using the modeltest-ng specified best model for each partition with 500 bootstrap replicates.

The generated trees were visualised in FigTree v1.4.3 (http://tree.bio.ed.ac.uk/software/figtree/).

### Morphological characterisation

Culture morphology was assessed on PDA (Nissui Pharmaceutical Co., Ltd., Tokyo, Japan), oatmeal agar (OA, Difco) and synthetic nutrient-poor agar (SNA^56^) plates. Autoclaved filter paper and double-autoclaved stems of *Anthriscus sylvestris* were placed on the surface of SNA plates. Plates were inoculated with 8-mm-diameter mycelia-grown agar plugs from the edge of actively growing cultures. Cultures were incubated at 24 °C under near UV light with 12 h photoperiod for 10 d. Colony colours were rated after 7 d using the Rayner colour chart^57^. Growth rates were estimated after 7 and after 10 d. Structures were observed after 10 d. For formation of appressoria, conidial suspensions were incubated on SNA or OA coated glass slides with a plastic cover slip in 100% humidity at 24 °C in the dark for 24 h. Fungal structures were observed using a Leica M165FC dissecting microscope (DM) and an Olympus BX51 microscope with differential interference contrast (DIC) optics. Samples for DIC were mounted directly in lactic acid. Measurements were made for at least 30 structures each.

### Pathogenicity tests and observation of the infection process

Perilla plants (Aka Chirimen Shiso, Takii & Co., Ltd.) were cultivated on sterile soil in 12 h white light/12 h dark at 24 °C for three weeks before infections. For observations of trypan blue-stained invasive hyphae, approximately 100 µL of a conidia suspension of strain JCM 31818 (1 × 10^5^ conidia/mL) was dropped onto the abaxial side of each detached perilla leaf. Then, pieces of 1.5 cm × 1 cm nylon mesh with 100 µm pores were placed on to conidial droplets to ensure even distribution of the conidial suspension on the surface of the leaf. Inoculated leaves were incubated in petri dishes for 40 and 60 hpi (hours post inoculation) at 24 °C under white light with 12 h light/12 h dark photoperiod and 100% humidity in a plastic dish with autoclaved filter paper moistened by sterilised water. For trypan blue staining, infected perilla leaves were boiled in 1 ml of alcoholic lactophenol (ethanol: phenol: glycerol: lactic acid: water (4:1:1:1:1, v/v/v/v/v)) containing 0.1 mg/ml trypan blue for 10 min at 95 °C and left overnight at room temperature. Boiled leaves were destained with chloral hydrate solution (500 g chloral hydrate + 200 ml water) overnight. Stained leaves were mounted in 60% glycerol solution and observed with DIC under a microscope. Further, for observation of setae, conidia and appressoria in/on *P. frutescens* leaves, intact three-week-old plants grown on sterile soil were sprayed with 5 × 10^5^ conidia/mL conidial solution. Leaves were detached just prior to observation and mounted directly in lactic acid before DIC imaging and measurements. For pathogenicity tests, intact three-week-old plants grown on sterile soil were sprayed with 5 × 10^5^ conidia/mL conidial solution. As negative controls, mock-treated plants were sprayed with water. Plants were maintained at 24 °C in 12 h white light/12 h dark conditions in 100% relative humidity and assessed for anthracnose lesions at two weeks post-inoculation. At least four plants were tested for each treatment in three independent experiments.

For pathogenicity tests of *A. thaliana*, the first three fully expanded leaves from four-week-old plants grown under short day conditions (8 h light/16 h dark) at 21 °C were inoculated 5 × 10^5^ conidia/ml conidial suspensions of strain JCM 31818 from perilla or *C. higginsianum* strain MAFF 305635 from *Brassica rapa*. Each leaf was inoculated with one 5 μl droplet of prepared conidial suspension. Infected plants were maintained at 100% humidity under the same light and growth conditions as perilla plants. Images of infected leaves were captured 6 d after inoculation using a Canon EOS-M camera and lesion areas were determined using ImageJ^58^. Experiments were repeated twice using eight plants per ecotype per experiment. Lesion area size distributions were tested for significant differences using Mann-Witney *U* tests. For both *P. frutescens* and *A. thaliana* pathogenicity tests, leaves were only detached from intact plants just prior to imaging.

### DNA extraction and genome sequencing

For sequencing and assembly of the JCM 31818 genome, genomic DNA was extracted and sequenced as previously described^17^. In brief, PD broth (BD Biosciences, Franklin Lakes, NJ, USA) was inoculated with hyphae from a growing colony. After incubating for 3 d at 24 °C under dark conditions, the mycelium was harvested and ground in liquid nitrogen and then the genomic DNA was extracted using CTAB buffer and 100/G genomic tips (QIAgen, Hilden, Germany) as previously described^53^. DNA from MAFF 240106 was extracted using the QIAgen genomic DNeasy kit according to the manufacturer’s instructions. Two differently sized insert libraries, 150 bp and 500 bp, were prepared using the Illumina TruSeq PCR-free DNA sample prep kit (Illumina) and sequenced to generate 100 bp paired-end reads with an Illumina HiSeq 2000 sequencing system (RIKEN Omics Science Center, Yokohama, Japan).

### Genome assembly and annotation

Low quality reads were trimmed using TrimGalore wrapper with cutadapt (v1.2.1) and fastqc (v0.11.7). Sequences were assembled using Megahit^59^ followed by scaffolding using the SSPACE-Standard-3.0 scaffolder (Baseclear). The assembly was assessed using quast v4.5^60^ and BUSCO v3.0 using the sordariomyceta_odb9 dataset^61^. The size of the genome was estimated by kmer analysis using jellyfish v1.14^62^ as previously described^63^. Genes were predicted with the MAKER v 2 pipeline^64^ after optimizing Augustus v 3.3^65^ gene model parameters by running the BUSCO pipeline^61^ with the--long option to identify *C. shisoi* homologs of 3,659 sordariomycete conserved proteins; training Genemark-ES (v3.51)^66^ on the *C. shisoi* genome using the option to run the program using the branch point model for fungal gene predictions; and including proteins from *C. higginsianum*^67^ as additional evidence for gene model support.

### Orthogroup identification

All predicted proteins from *C. graminicola*^18^, *C. higginsianum*^67^, *C. incanum*^17^ and *C. tofieldiae*^37,68^ were analysed using OrthoFinder v 2.2.6^69^ with the default settings. For identification of secreted protein orthogroups, Deeploc v1.0 was utilised to predict the localisations of proteins from each species^70^. Then, OrthoFinder was run on the predicted secreted proteins to identify orthogroups within the secreted protein sequences. For analysis of dN/dS values of secreted protein-encoding gene sequences, the genes of secreted proteins grouped together by OrthoFinder were aligned using PRANK^71^ to produce codon alignments using default settings. Codon alignments were then analysed using the yn00 model^72^ implemented in the PAML suite of programs^73^. Conservation plots were drawn using the UpsetR package^74^ in R^75^. GO terms were assigned to *C. shisoi* sequences using Trinotate^76^ and enrichment of GO terms in selected groups was tested using the hypergeometric test in the GOstats package^77^ and applying the Benjamini-Hochberg multiple test correction on P-values using R.

## Supplementary information

Supplementary figures S1–17

Supplementary Tables S1–6

## Data Availability

*C. shisoi* sequences used for phylogenetic analyses are deposited under GenBank accession numbers MH660928-MH660937. The whole genome shotgun sequences were deposited in DDBJ/ENA/GenBank under BioProject PRJNA431477 with accession number PUHP00000000. In this study, version PUHP01000000 is described.
