# Supplementary material for: Colletotrichum shisoi sp. nov., an anthracnose pathogen of Perilla frutescens in Japan: molecular phylogenetic, morphological and genomic evidence
Source: Sci Rep. 2019 Sep 16;9:13349. doi: 10.1038/s41598-019-50076-5 (PMC6746953; doi:10.1038/s41598-019-50076-5)
Supplement: Supplementary file 1 — Supplementary figures S1–17 [file 41598_2019_50076_MOESM1_ESM.pdf]

Supplementary Information

Article in Scientific Reports

Colletotrichum shisoi **sp. nov., an anthracnose pathogen of** ***Perilla frutescens*** **in Japan: molecular phylogenetic, morphological and genomic evidence**

Gan P., Tsushima A., Hiroyama R., Narusaka M., Takano, Y., Narusaka Y., Kawaradani M., Damm U. and Shirasu K.

Supplementary Figures 1-17

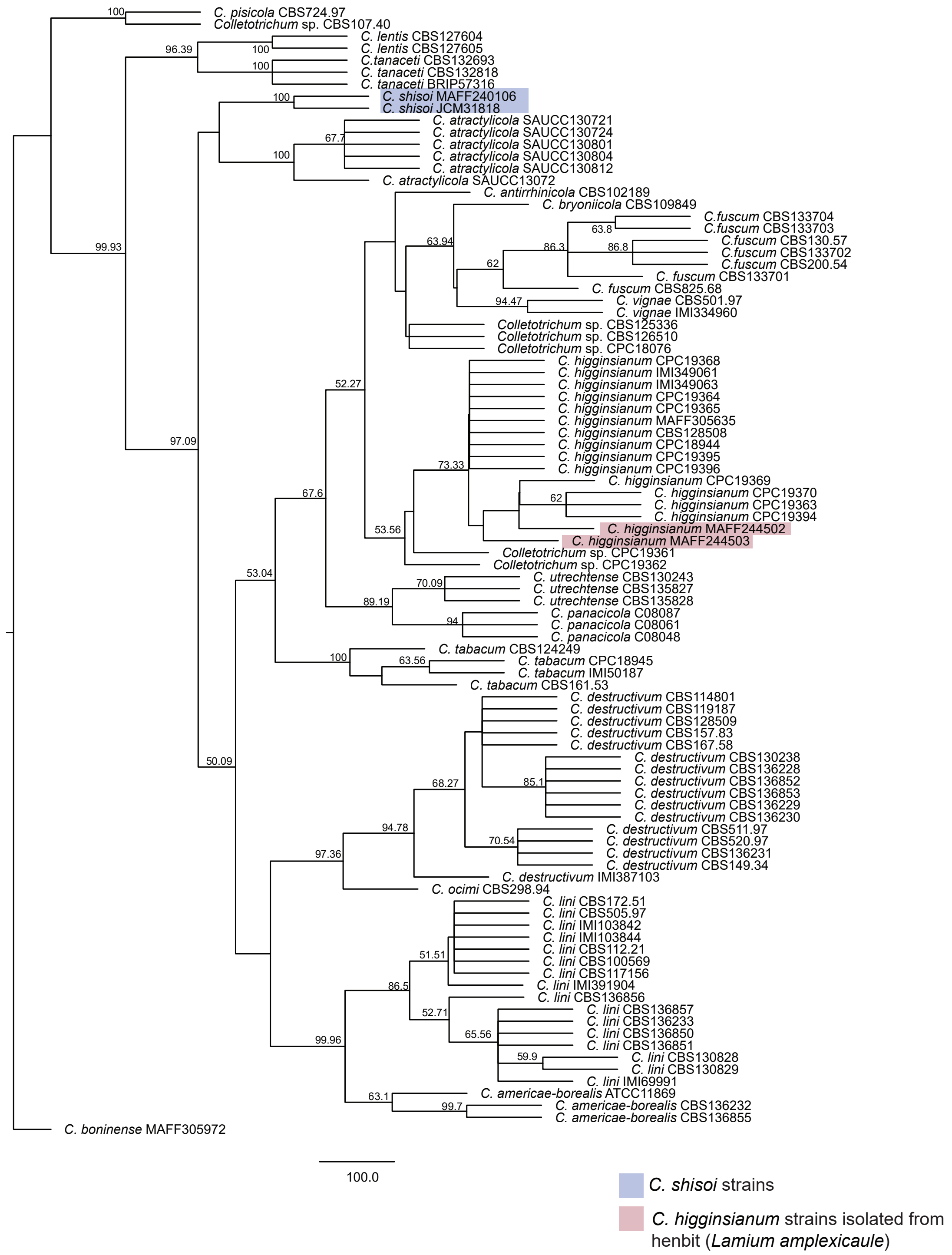

Supplementary Fig. 1

The first of 64 equally most parsimonious trees generated by a heuristic search of the combined ITS, *GAPDH*, *CHS-1, ACT* and *TUB2* sequence alignment from the *Colletotrichum destructivum* species complex using *Colletotrichum boninense* MAFF 305972 as an outgroup. Values at nodes are percentages of bootstrap support values out of 1,000. Only bootstrap values with greater than 50 % support are indicated.

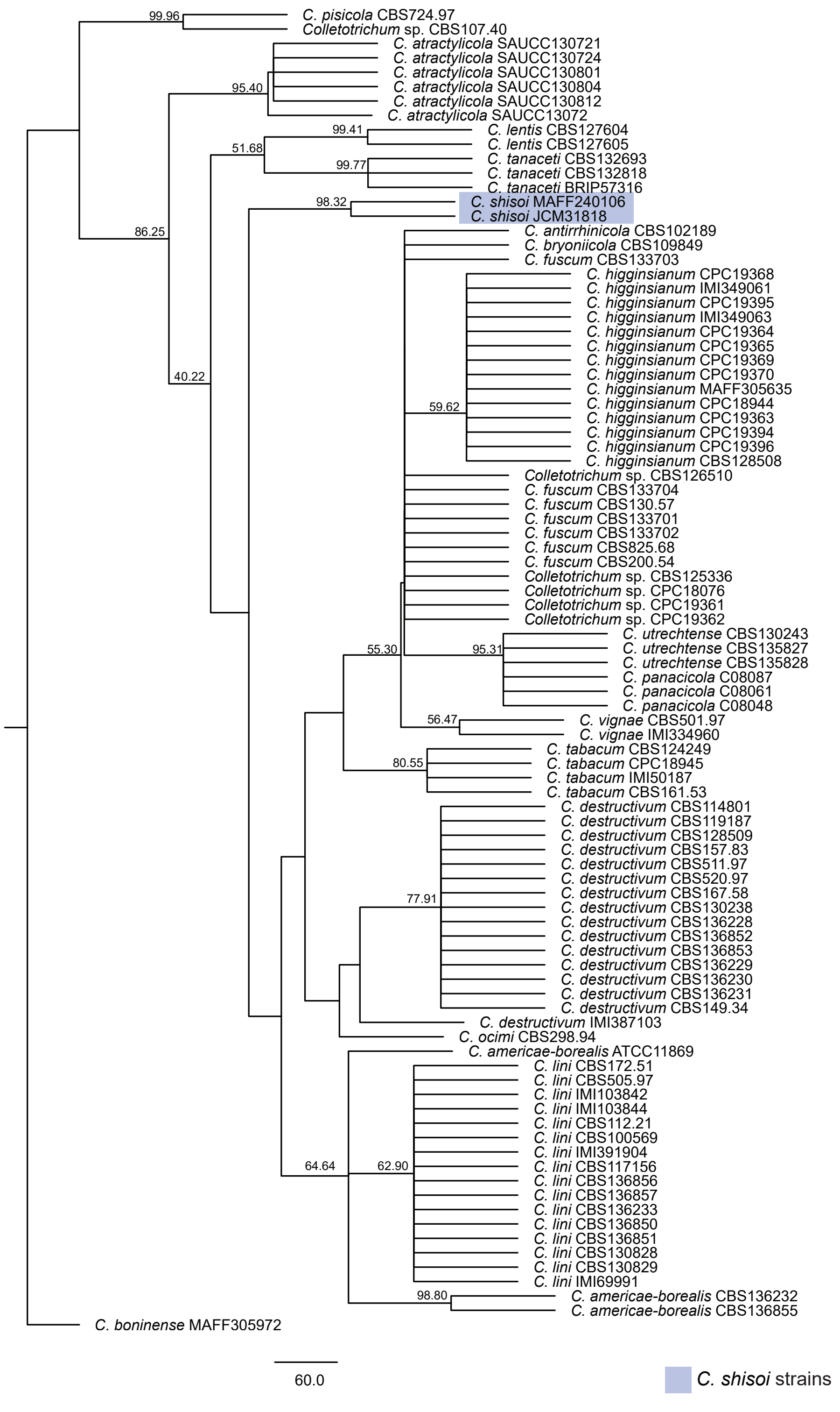

Supplementary Fig. 2

One of 11 equally most parsimonious trees generated by a heuristic search of the actin (*ACT*) sequence alignment from the *Colletotrichum destructivum* species complex using *Colletotrichum boninense* MAFF 305972 as an outgroup. Values at nodes are percentages of bootstrap support values out of 1000. Only bootstrap values with greater than 50 % support are indicated.

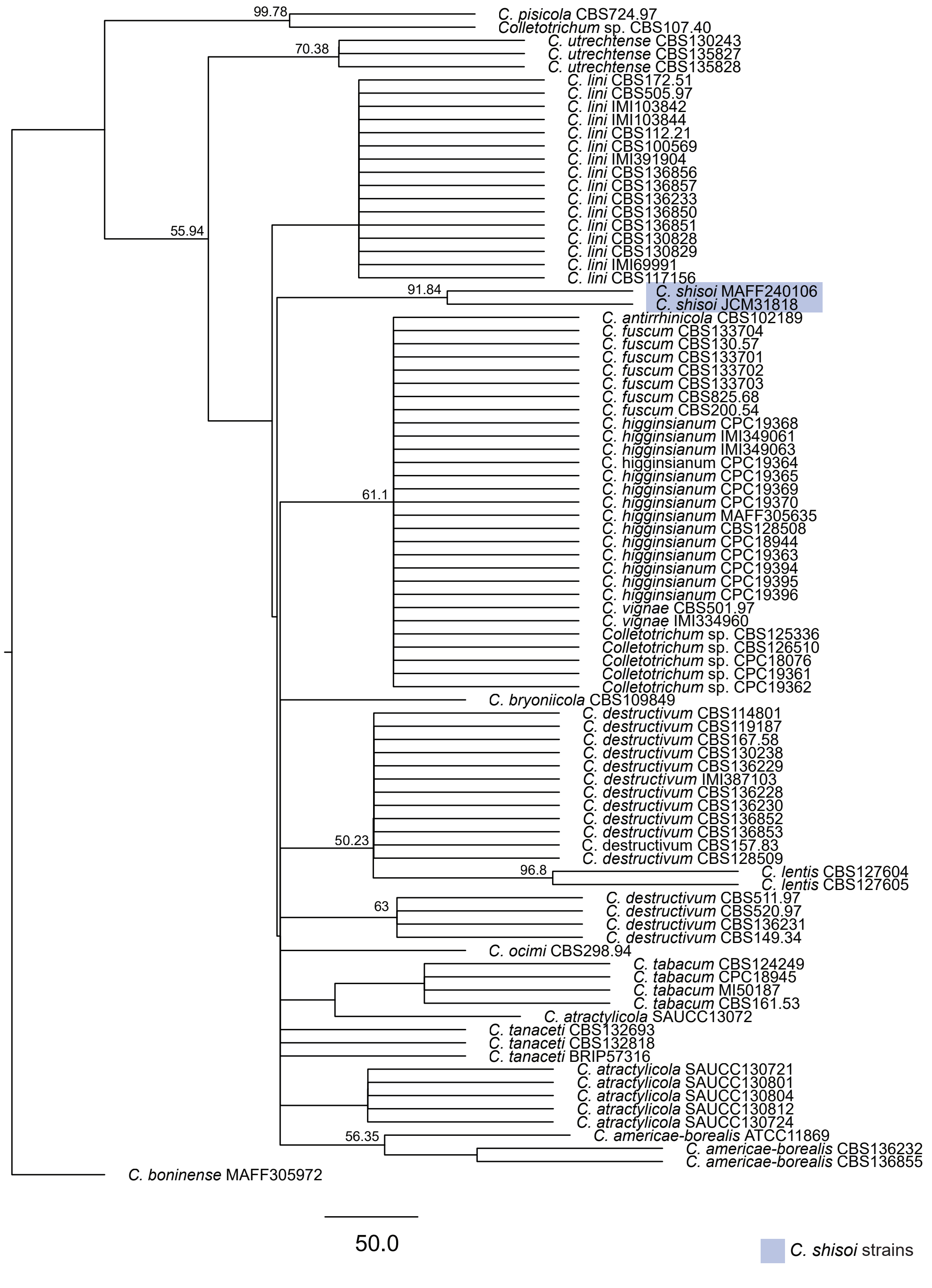

Supplementary Fig. 3
One of 5 equally most parsimonious trees generated by a heuristic search of the chitin synthase 1 (*CHS-1*) sequence alignments from the *Colletotrichum destructivum* species complex using *Colletotrichum boninense* MAFF 305972 as an outgroup. Values at nodes are percentages of bootstrap support values out of 1000. Only bootstrap values with greater than 50 % support are indicated.

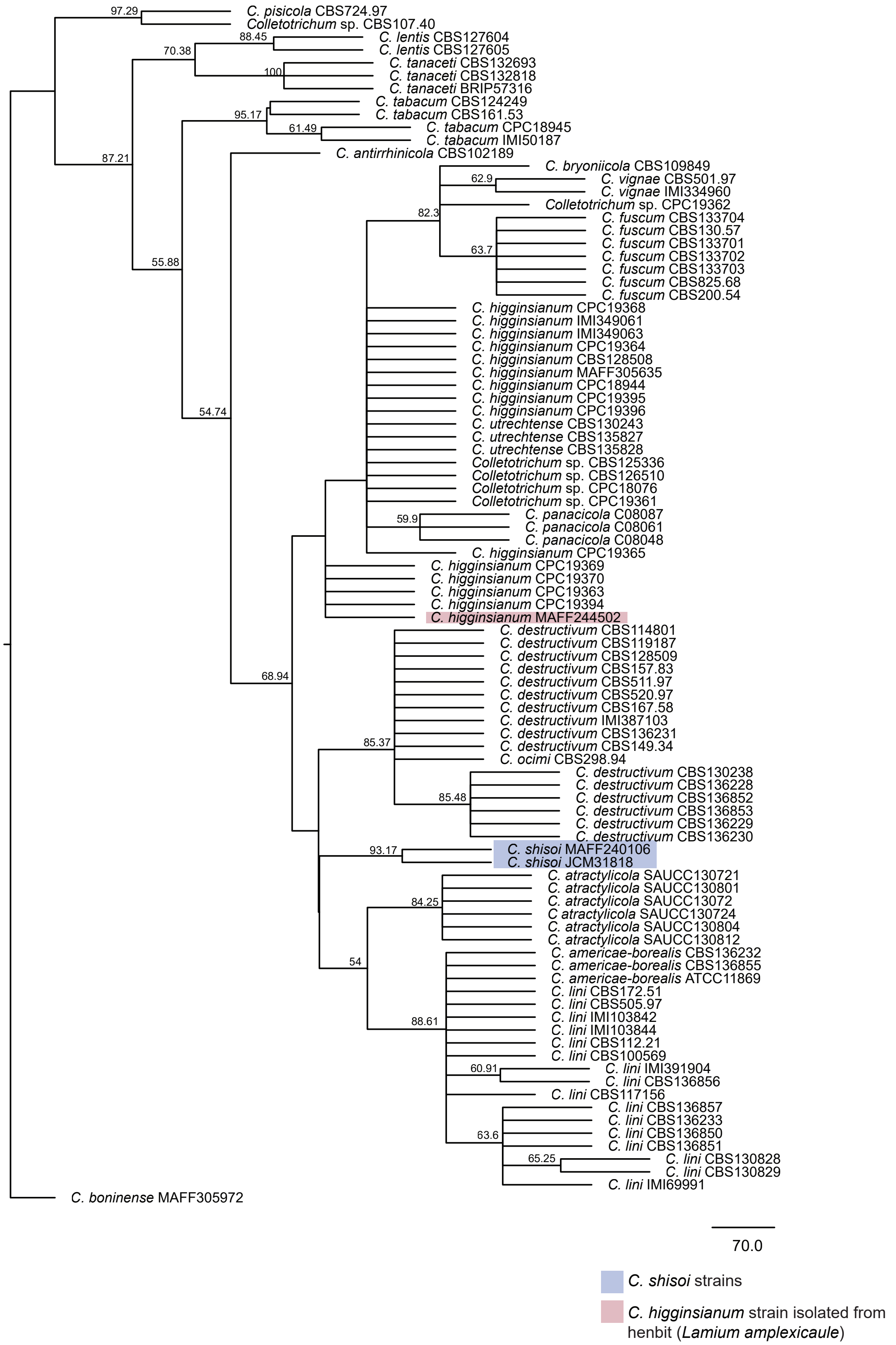

Supplementary Fig. 4

One of 4 equally most parsimonious trees generated by a heuristic search of the glyceraldehyde-3-phosphate dehydrogenase (*GAPDH*) sequences of the *Colletotrichum destructivum* species complex using *Colletotrichum boninense* MAFF 305972 as an outgroup. Values at nodes are Bayesian posterior probabilities.

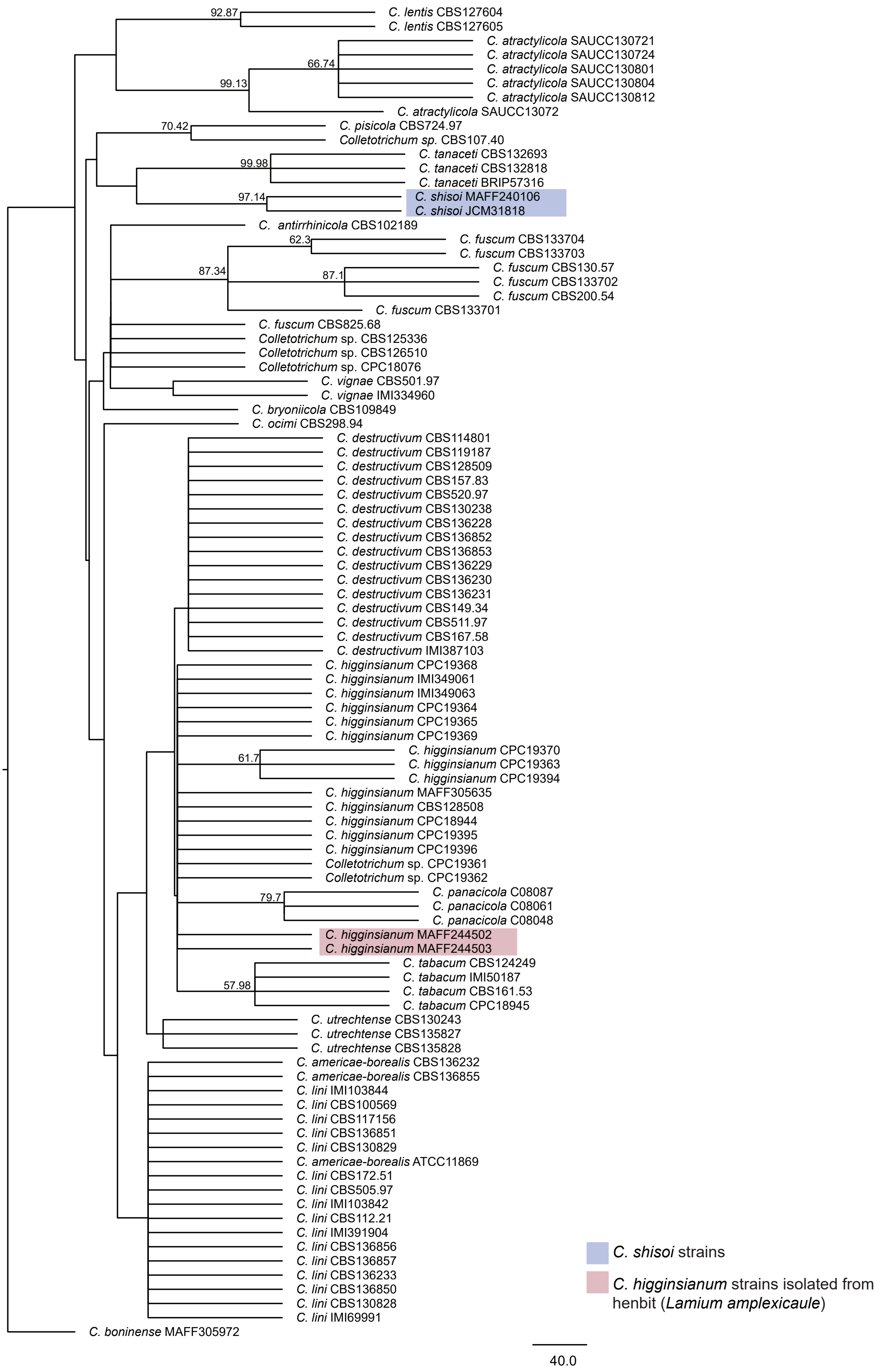

Supplementary Fig. 5

One of 100 equally most parsimonious trees generated by a heuristic search of the internal transcribed spacers (ITS) sequences of the *Colletotrichum destructivum* species complex using *Colletotrichum boninense* MAFF 305972 as an outgroup. Values at nodes are Bayesian posterior probabilities.

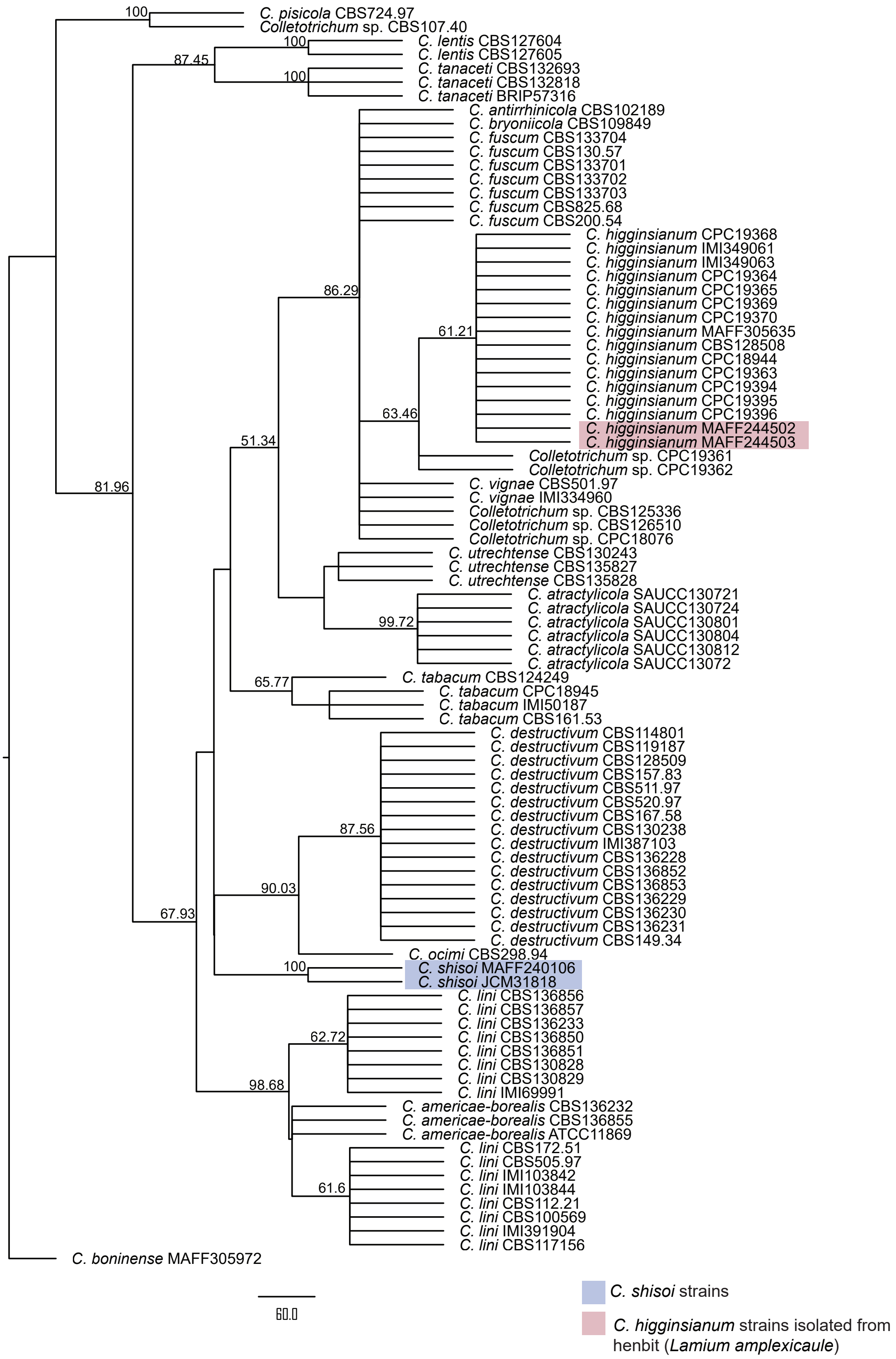

Supplementary Fig. 6

One of 14 equally most parsimonious trees generated by a heuristic search of the beta-tubulin (*TUB2*) sequences of the *Colletotrichum destructivum* species complex using *Colletotrichum boninense* MAFF 305972 as an outgroup. Values at nodes are Bayesian posterior probabilities.

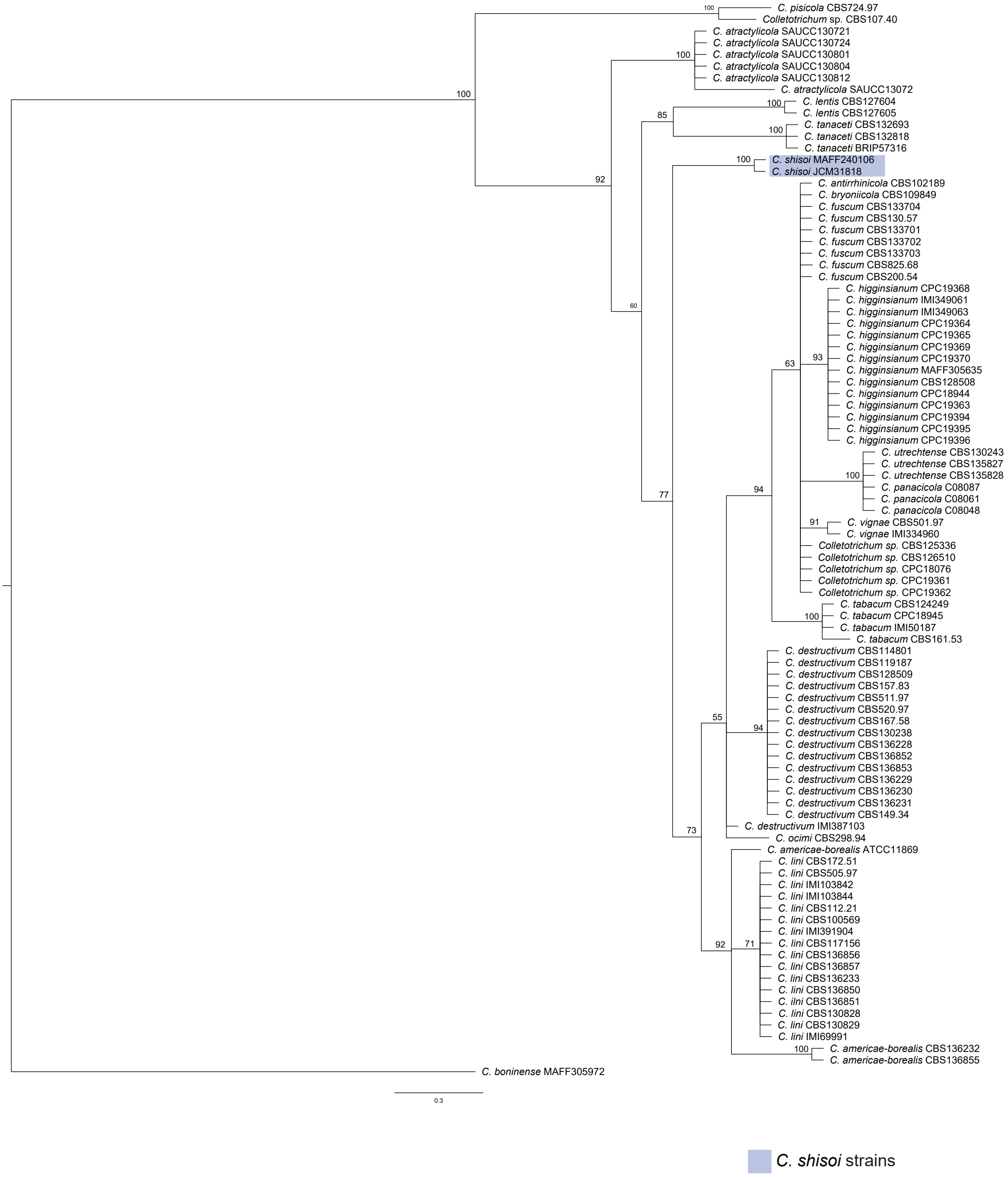

Supplementary Fig. 7

Phylogenetic tree based on actin (*ACT*) sequences of the *Colletotrichum destructivum* species complex using *Colletotrichum boninense* MAFF 305972 as an outgroup. Values at nodes are Bayesian posterior probabilities.

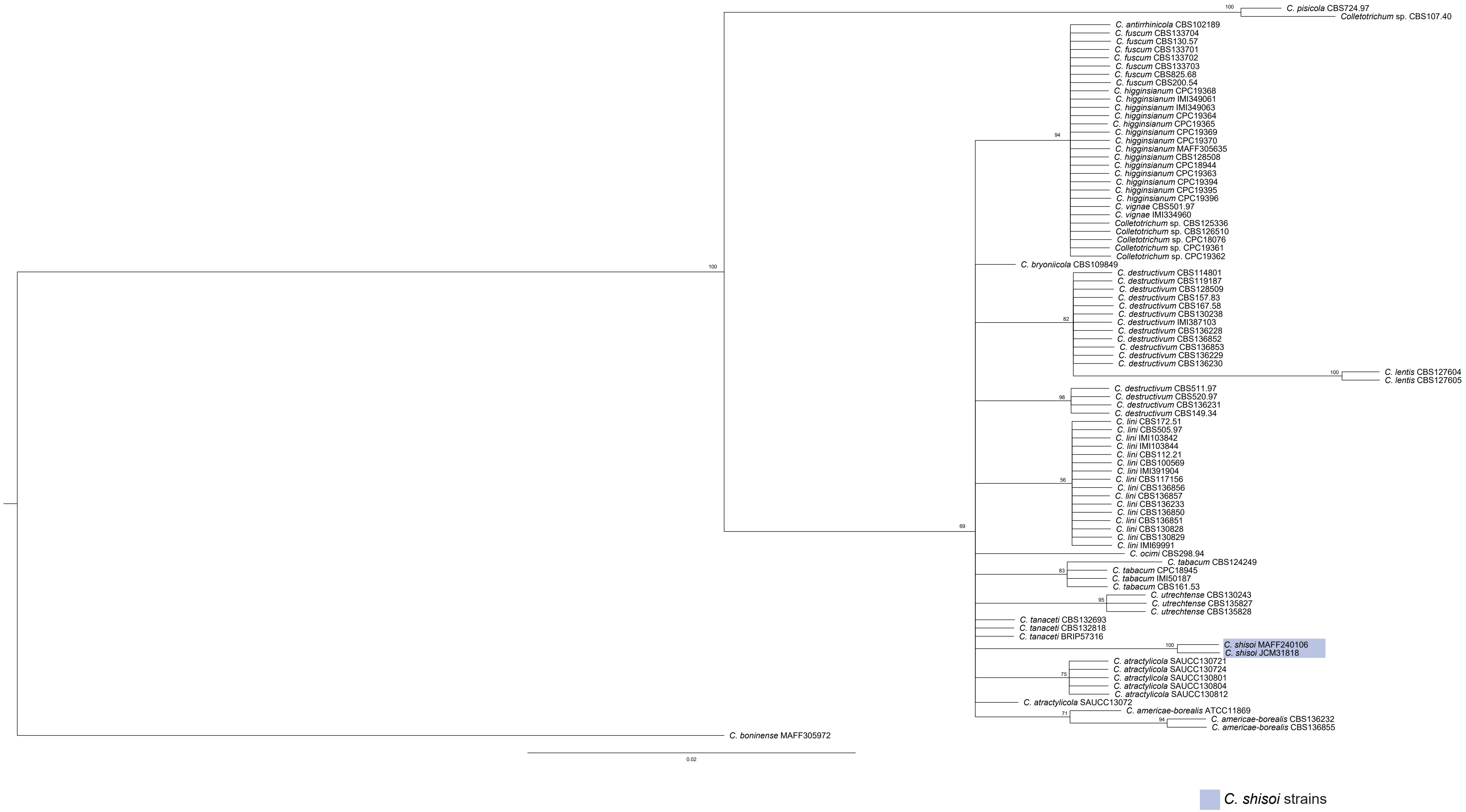

Supplementary Fig. 8
Phylogenetic tree based on chitin synthase 1 (*CHS-1*) sequences of the *Colletotrichum destructivum* species complex using *Colletotrichum boninense* MAFF 305972 as an outgroup. Values at nodes are Bayesian posterior probabilities.

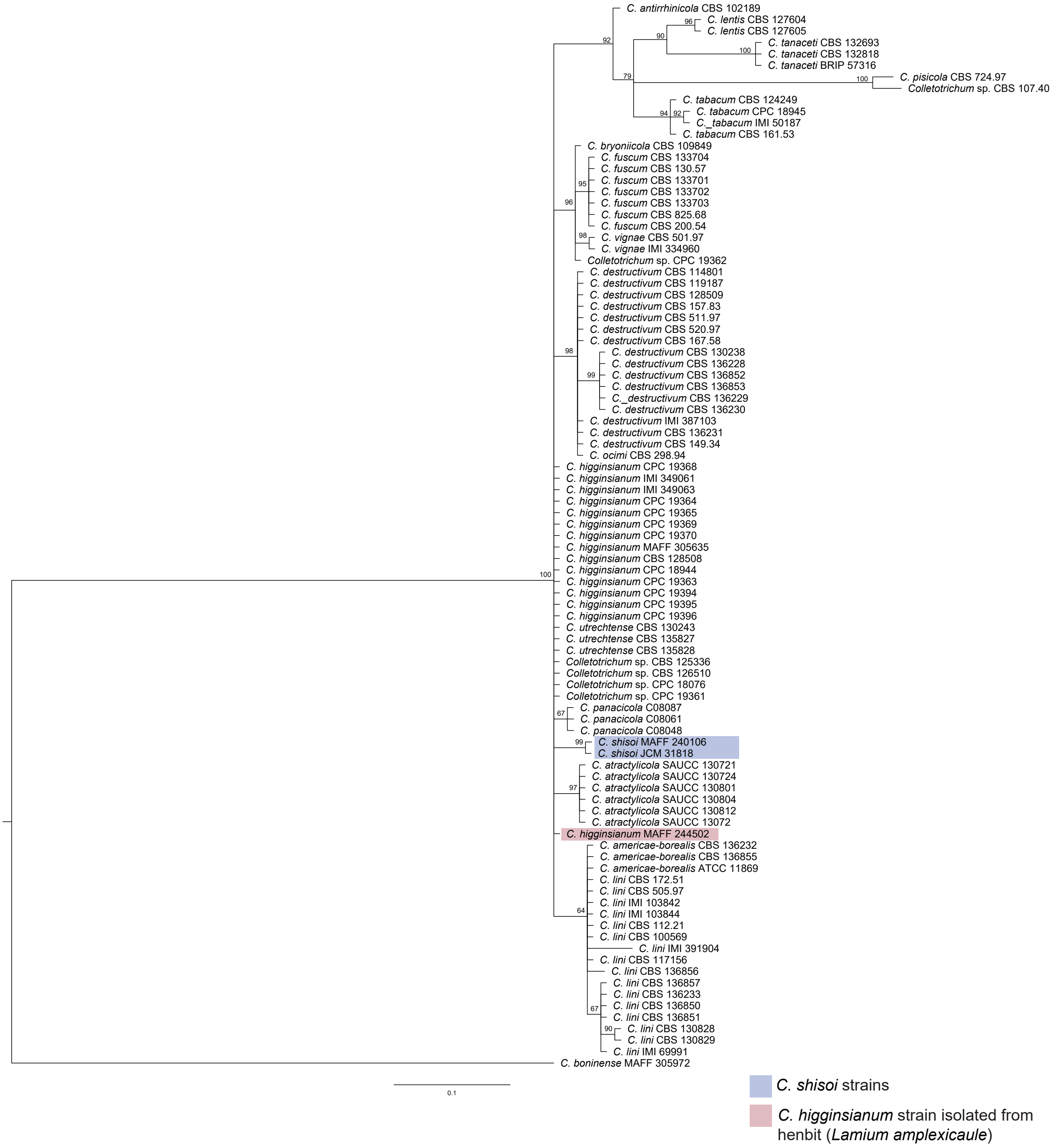

Supplementary Fig. 9

Phylogenetic tree based on glyceraldehyde-3-phosphate dehydrogenase (*GAPDH*) sequences of the *Colletotrichum destructivum* species complex using *Colletotrichum boninense* MAFF 305972 as an outgroup. Values at nodes are Bayesian posterior probabilities.

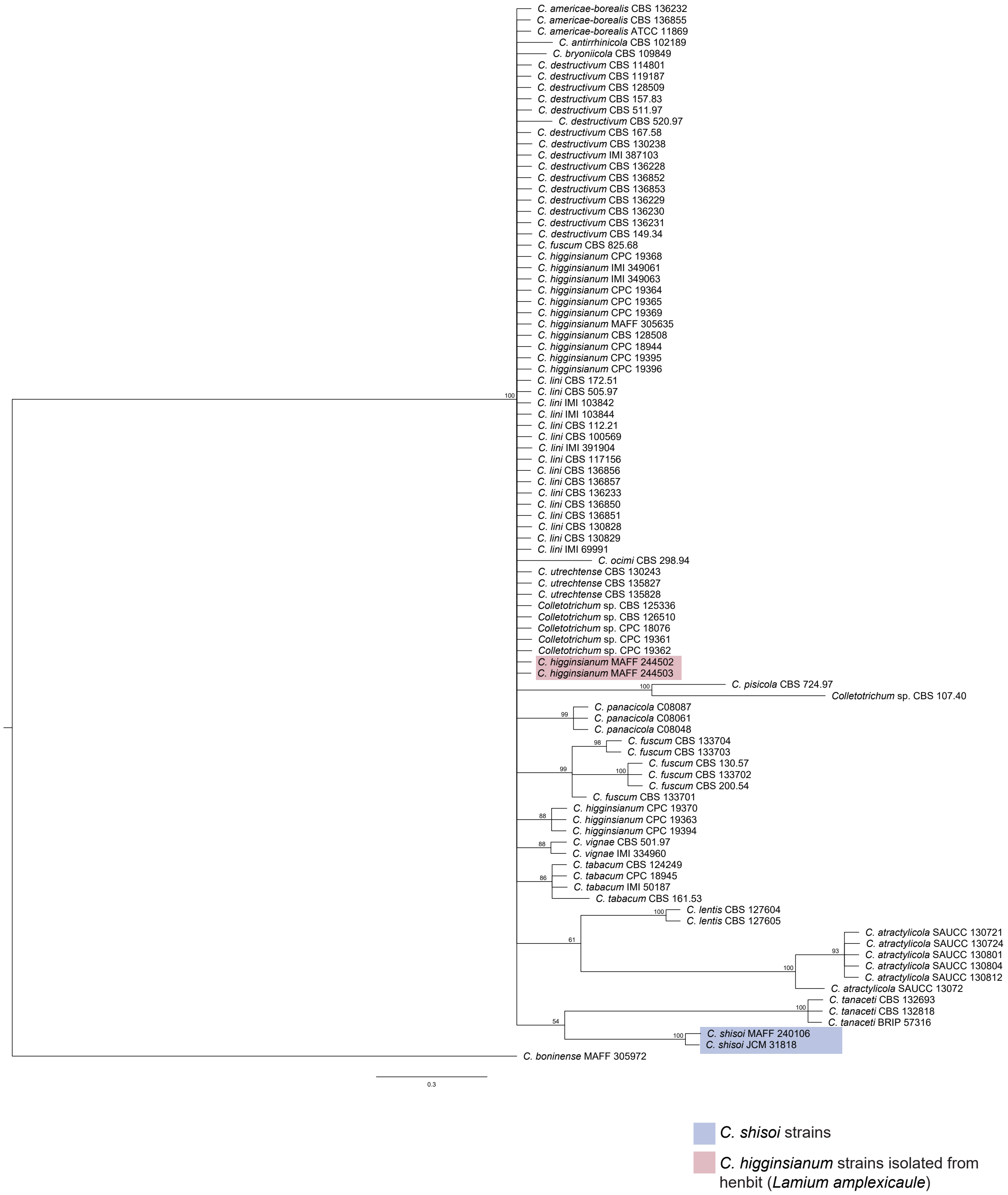

Supplementary Fig. 10
Phylogenetic tree based on internal transcribed spacers (ITS) sequences of the *Colletotrichum destructivum* species complex using *Colletotrichum boninense* MAFF 305972 as an outgroup. Values at nodes are Bayesian posterior probabilities.

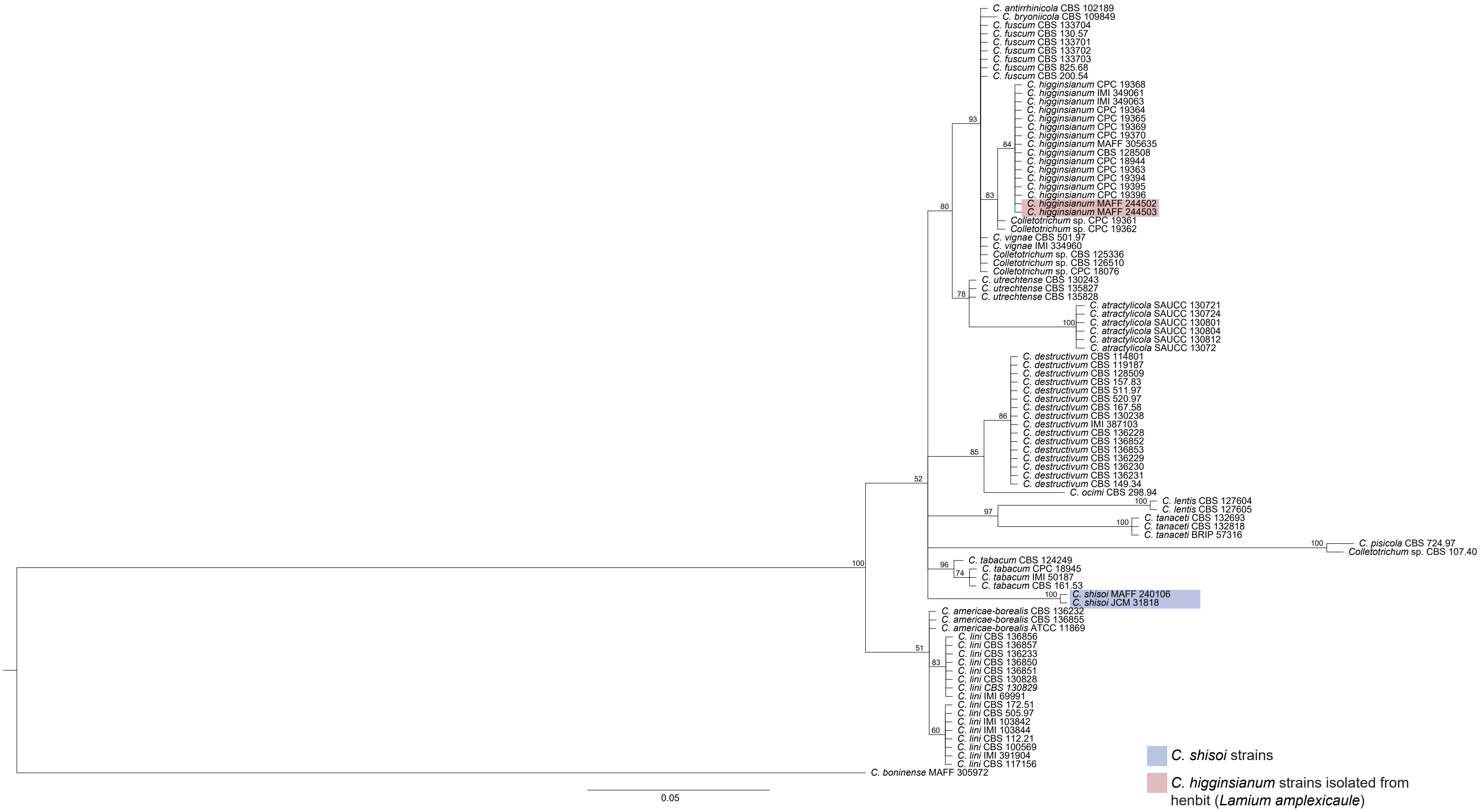

Supplementary Fig. 11
Phylogenetic tree based on beta-tubulin (*TUB2*) sequences of the *Colletotrichum destructivum* species complex using *Colletotrichum boninense* MAFF 305972 as an outgroup. Values at nodes are Bayesian posterior probabilities.

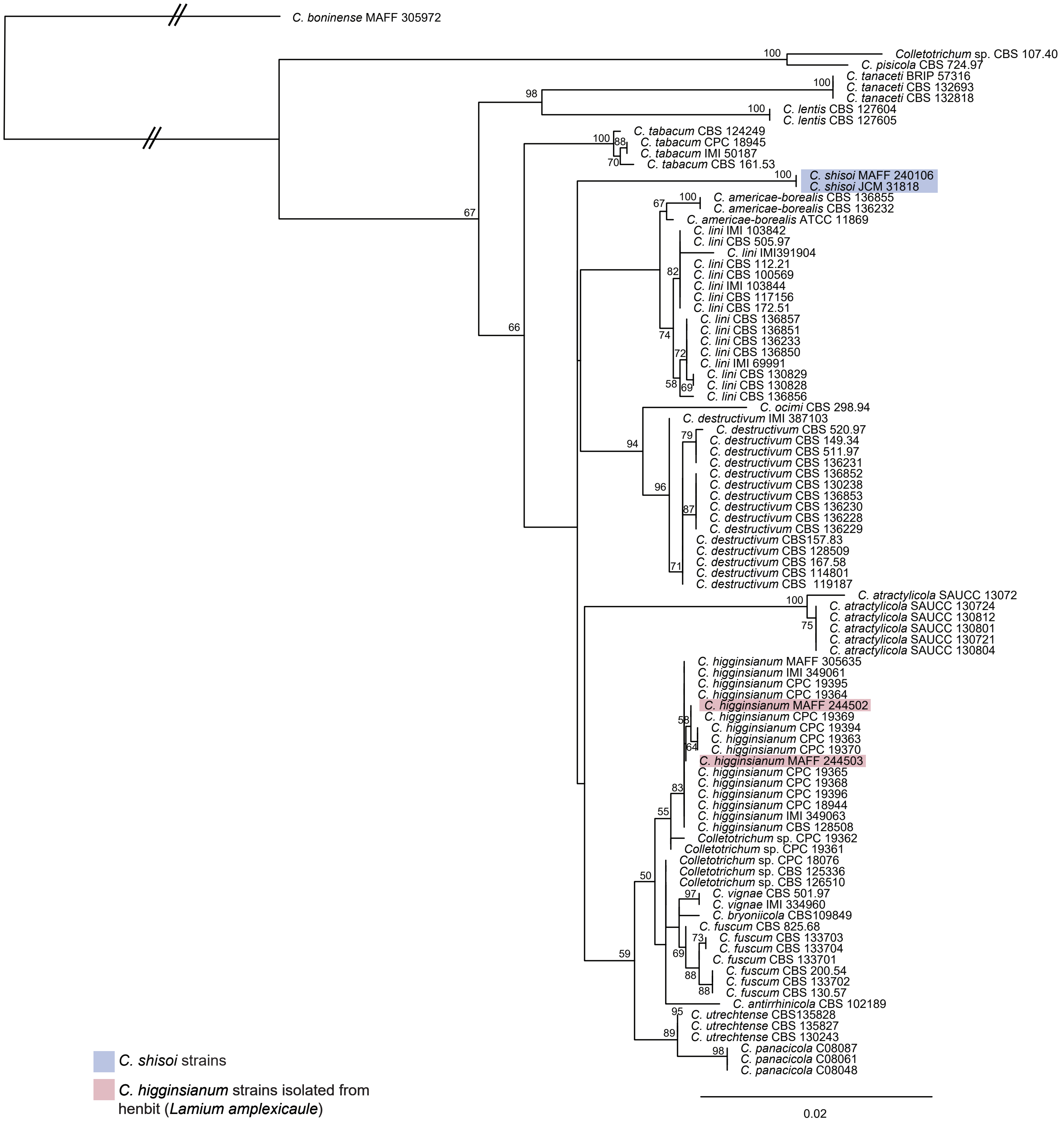

Supplementary Fig. 12

Multi-locus maximum likelihood tree based on ITS, *GAPDH, CHS-1, ACT* and *TUB2* sequences of the *Colletotrichum destructivum* species complex using *Colletotrichum boninense* MAFF 305972 as an outgroup. Values at nodes are percentages of bootstrap support values out of 1,000. Only bootstrap values with greater than 50 % support are indicated. Branches crossed with double bars have been shortened by five-fold.

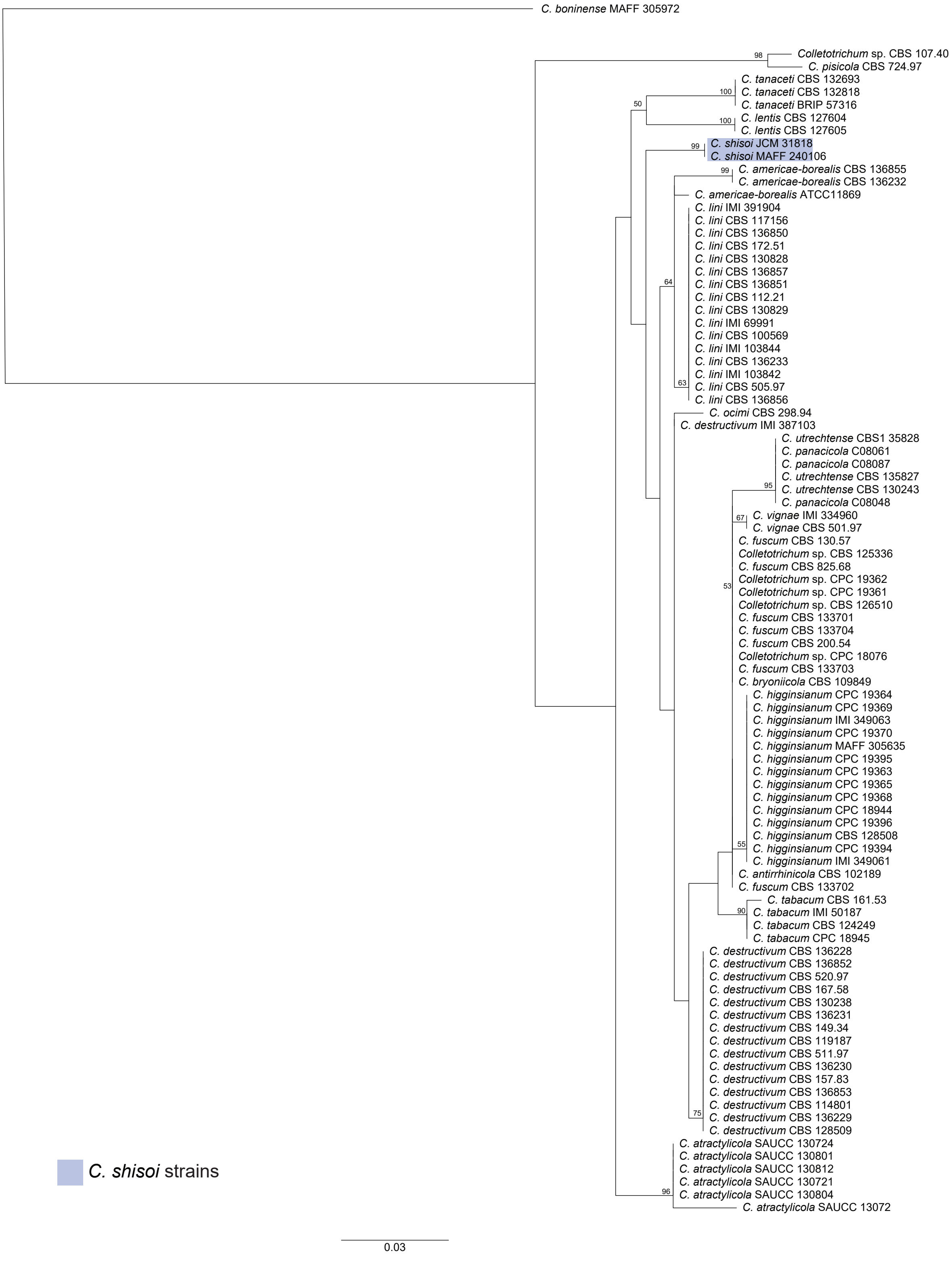

Supplementary Fig. 13
Maximum likelihood tree based on actin (*ACT*) sequences of the *Colletotrichum destructivum* species complex using *Colletotrichum boninense* MAFF 305972 as an outgroup. Values at nodes are percentages of bootstrap support values out of 1,000. Only bootstrap values with greater than 50 % support are indicated.

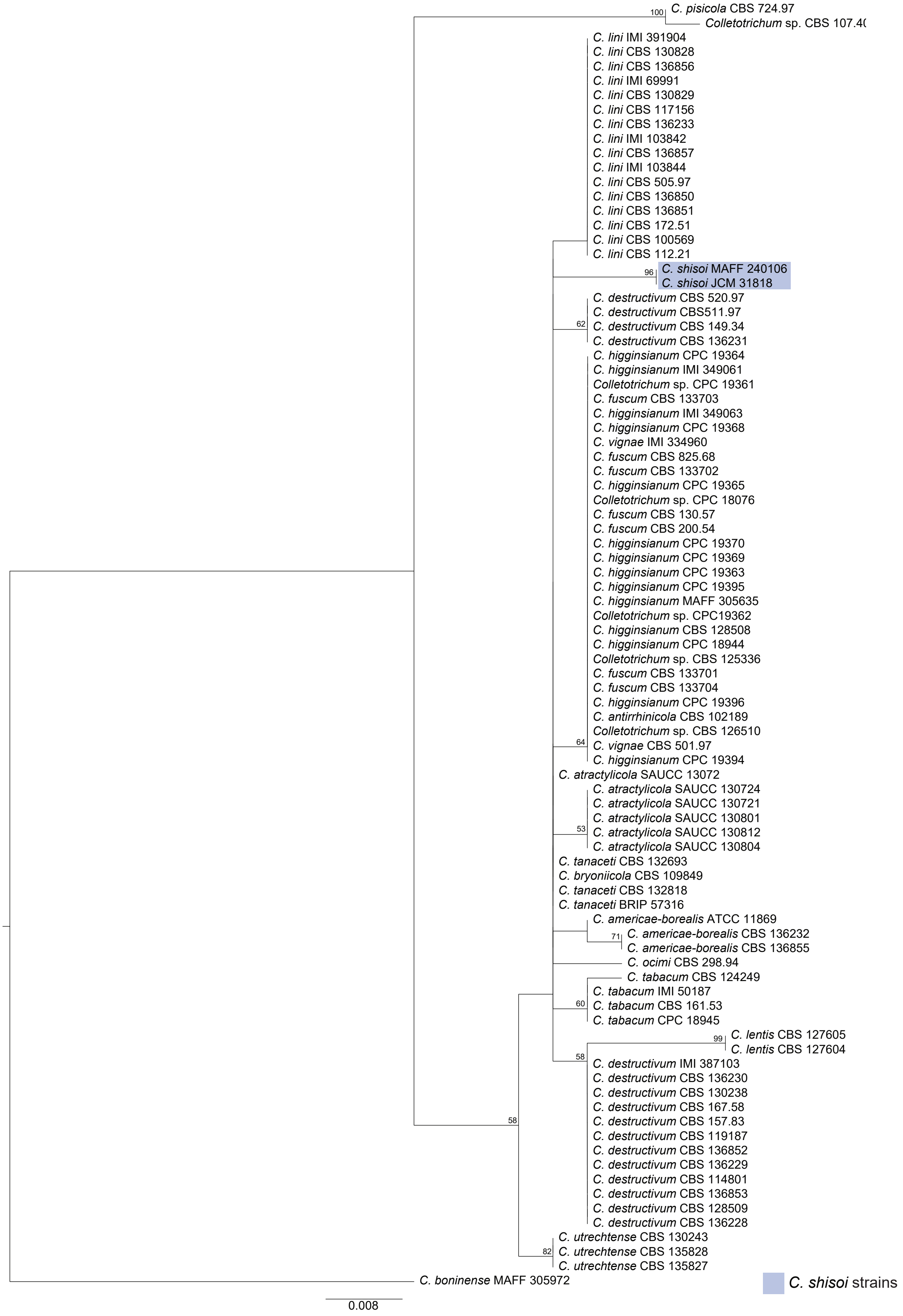

Supplementary Fig. 14

Maximum likelihood tree based on chitin synthase 1 (*CHS-1*) sequences of the *Colletotrichum destructivum* species complex using *Colletotrichum boninense* MAFF 305972 as an outgroup. Values at nodes are percentages of bootstrap support values out of 1,000. Only bootstrap values with greater than 50 % support are indicated.

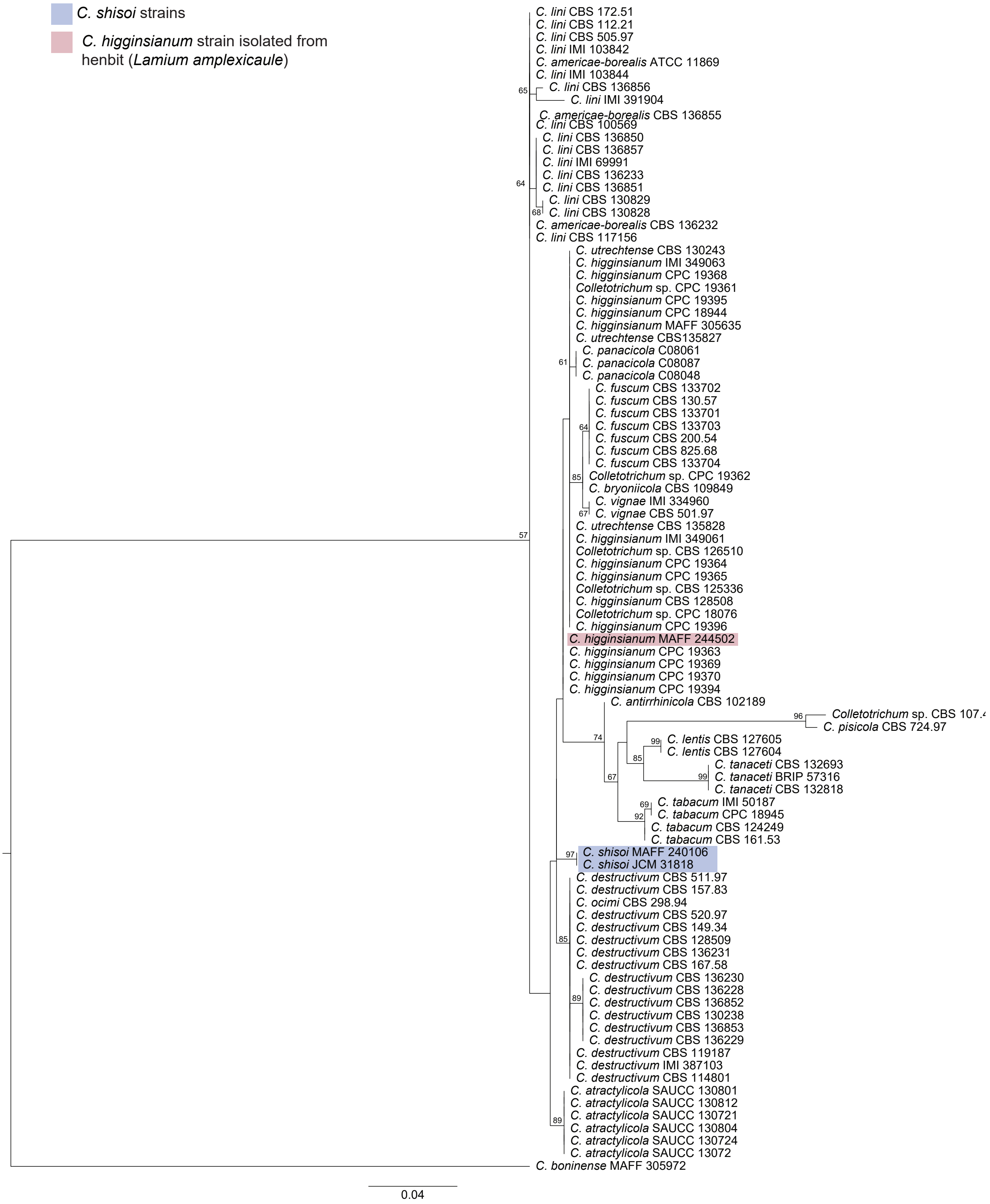

Supplementary Fig. 15

Maximum likelihood tree based on glyceraldehyde-3-phosphate dehydrogenase (*GAPDH*) sequences of the *Colletotrichum destructivum* species complex using *Colletotrichum boninense* MAFF 305972 as an outgroup. Values at nodes are percentages of bootstrap support values out of 1,000. Only bootstrap values with greater than 50 % support are indicated.

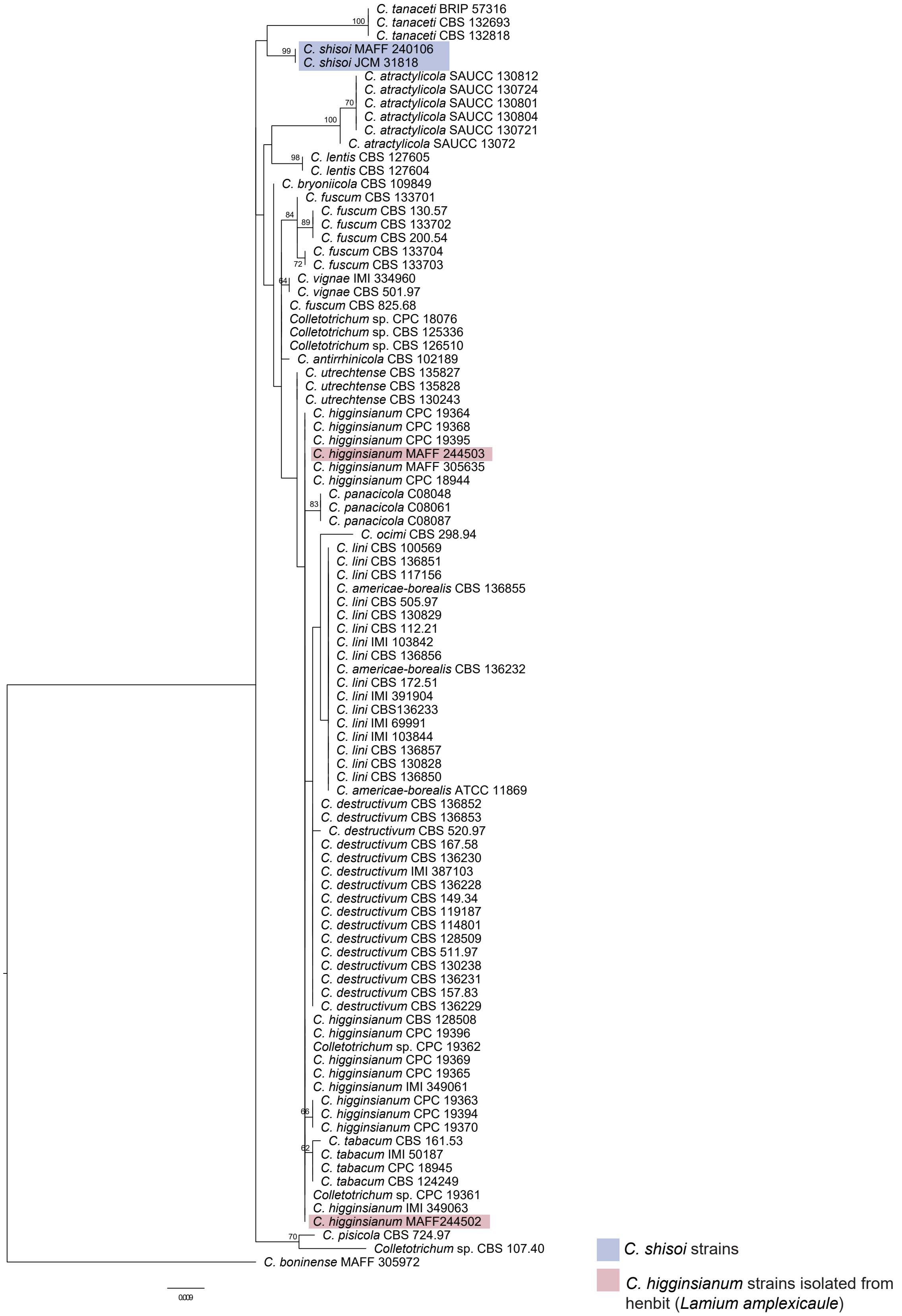

Supplementary Fig. 16

Maximum likelihood tree based on internal transcribed spacers (ITS) sequences of the *Colletotrichum destructivum* species complex using *Colletotrichum boninense* MAFF 305972 as an outgroup. Values at nodes are percentages of bootstrap support values out of 1,000. Only bootstrap values with greater than 50 % support are indicated.

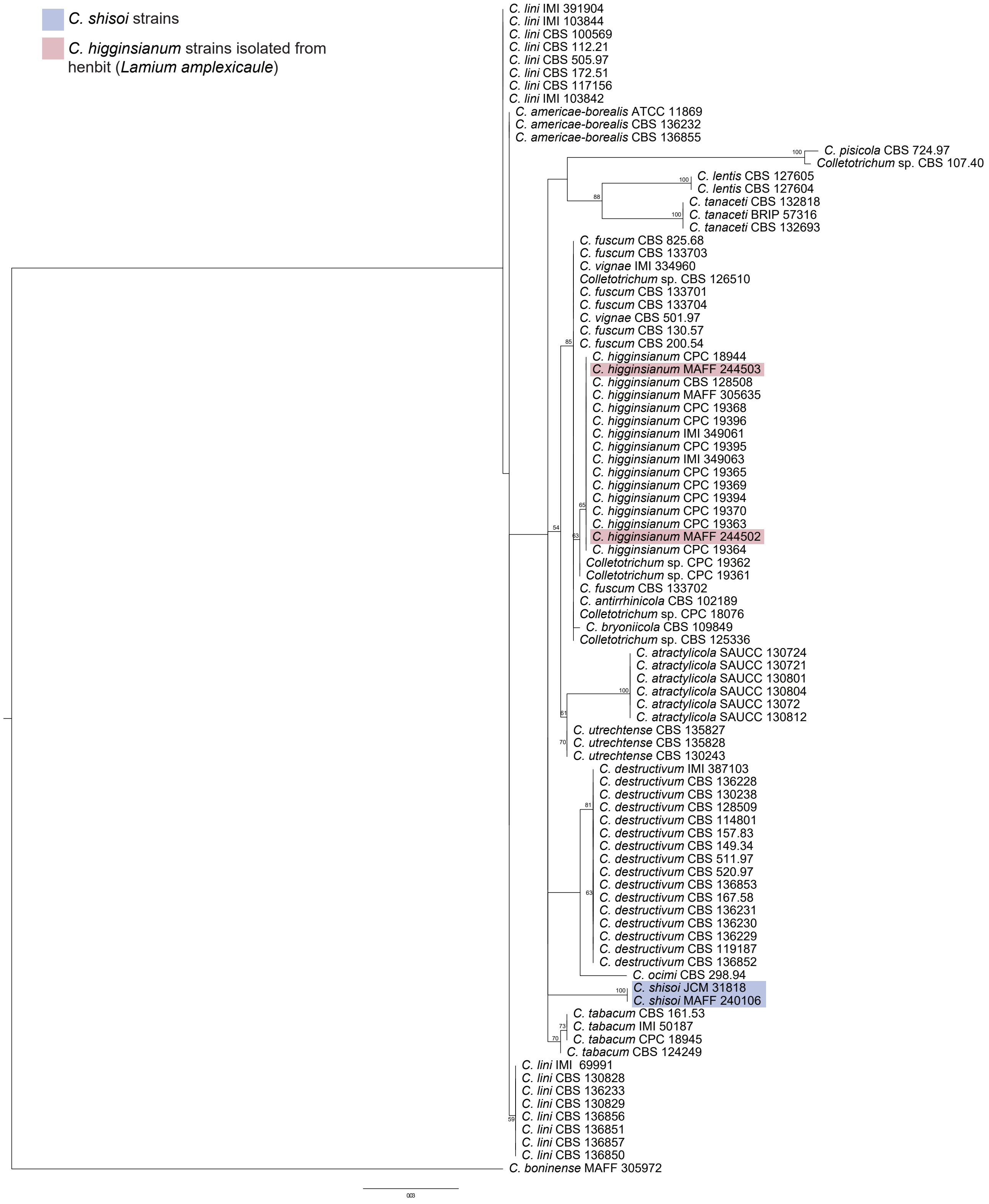

Supplementary Fig. 17

Maximum likelihood tree based on beta-tubulin (*TUB2*) sequences of the *Colletotrichum destructivum* species complex using *Colletotrichum boninense* MAFF 305972 as an outgroup. Values at nodes are percentages of bootstrap support values out of 1,000. Only bootstrap values with greater than 50 % support are indicated.
